# High-intensity interval training after stroke: a three-level random-effects meta-analysis with cluster-robust inference and exploratory dose-parameter signals

**DOI:** 10.3389/fneur.2026.1804120

**Published:** 2026-07-03

**Authors:** Xuanzi Zhang, Jie Yang, Juanjuan Hu, Zhiyuan Tan

**Affiliations:** 1 Department of Physical Education, Chengdu College of University of Electronic Science and Technology of China, Chengdu, China; 2 Faculty of Sport and Physical Education, University of Belgrade, Belgrade, Serbia

**Keywords:** 10-m walk test, 6-min walk test, balance, berg balance scale, blood pressure, cardiorespiratory fitness, exploratory analysis, GRADE

## Abstract

**Objective:**

To estimate the effects of high-intensity interval training (HIIT) on balance, walking outcomes, and physiological endpoints after stroke, and to generate hypotheses about whether training-load parameters may explain variability in intervention effects.

**Methods:**

We searched PubMed, Web of Science, Embase, Scopus, and the Cochrane Library from inception to December 31, 2025, for English-language randomized controlled trials of HIIT in post-stroke populations. Two reviewers independently screened records and extracted data. Risk of bias was assessed using RoB 2, and certainty of evidence was assessed using GRADE. Effect sizes were calculated as Hedges' g using between-group change scores. When change-score standard deviations were unavailable, they were imputed using a pre–post correlation of *r* = 0.5, with sensitivity analyses varying *r*. Effects were pooled using three-level random-effects models to accommodate dependent effect sizes. Statistical inference, including 95% confidence intervals and *p*-values, was based on cluster-robust variance estimation with small-sample correction. Meta-regression and subgroup analyses were conducted as exploratory, hypothesis-generating analyses of heterogeneity rather than confirmatory tests of training-load effects. The protocol was registered in PROSPERO (CRD42027809778).

**Results:**

Fourteen trials involving 717 participants were included. Pooled estimates suggested that HIIT may improve balance, as measured by the Berg Balance Scale, although the effect was small and the certainty of evidence was low (*ES* = 0.20, 95% CI 0.01 to 0.39, *p* = 0.039). HIIT may also improve walking endurance, as measured by the 6-min walk test, but the certainty of evidence was very low (*ES* = 0.41, 95% CI 0.22 to 0.61, *p* < 0.001). No statistically significant effect was observed for 10-meter walk test time (*ES* = 0.06, 95% CI−0.16 to 0.29, *p* = 0.579). Pooled estimates suggested a possible improvement in cardiorespiratory fitness, although the certainty of evidence was very low (*ES* = 0.36, 95% CI 0.05 to 0.66, *p* = 0.021). Effects on systolic blood pressure (*ES* = 0.05, 95% CI−0.25 to 0.36, *p* = 0.722) and diastolic blood pressure (*ES* = 0.25, 95% CI−0.05 to 0.56, *p* = 0.099) were not statistically significant. Evidence for stroke severity, assessed using the Scandinavian Stroke Scale, was sparse and uncertain (*ES* = 0.29, 95% CI−0.04 to 0.62, *p* = 0.084). Exploratory meta-regression and subgroup analyses identified preliminary signals of between-study variability, but these findings should be interpreted strictly as hypothesis-generating because of the small number of trials, limited outcome-specific effect sizes, and multiple comparisons. Overall, the certainty of evidence was low to very low across outcomes.

**Conclusions:**

Low- to very-low-certainty evidence suggests that HIIT may improve balance, walking endurance, and cardiorespiratory fitness after stroke, whereas effects on short-distance walking speed or gait control, blood pressure, and stroke severity remain uncertain. Apparent associations between training-load parameters and outcomes should not be interpreted as evidence of optimal HIIT prescriptions. These exploratory findings require confirmation in adequately powered randomized trials with standardized HIIT definitions, rigorous reporting of achieved intensity, and longer follow-up.

**Systematic review registration:**

https://www.crd.york.ac.uk/prospero/, identifier: CRD42027809778.

## 1 Introduction

Stroke is a major cause of long-term adult disability and is frequently associated with persistent impairments in balance, gait, and functional mobility. Balance impairment is one of the most common post-stroke deficits and is closely related to reduced postural control, gait instability, fear of falling, and increased fall risk, with important consequences for activities of daily living and quality of life (1). Although rehabilitation during the acute and early recovery phases can improve functional outcomes, many stroke survivors continue to experience residual balance and walking limitations in the subacute and chronic stages (2, 3). These persistent deficits may reflect neurological impairment, reduced cardiopulmonary fitness, lower-limb weakness, impaired sensorimotor integration, and insufficient training intensity or dose during rehabilitation (4, 5).

Post-stroke rehabilitation commonly includes task-specific physical therapy, balance training, gait training, and aerobic conditioning. Among these outcomes, balance is clinically important because it underpins safe standing, transfers, turning, walking, and community mobility (6). The Berg Balance Scale (BBS) is widely used to assess static and dynamic balance after stroke and provides a clinically interpretable measure of postural control during functional tasks. Therefore, in this review, balance assessed primarily by the BBS was defined as the primary outcome. Walking endurance, represented by the 6-min walk test (6MWT), and short-distance walking speed or gait-control performance, represented by the 10-meter walk test (10MWT), were considered key secondary functional outcomes. Cardiorespiratory fitness, blood pressure, and stroke severity were treated as exploratory physiological or clinical outcomes.

High-intensity interval training (HIIT), which alternates repeated bouts of relatively high-intensity exercise with recovery periods, has received increasing attention in stroke rehabilitation. Compared with moderate-intensity continuous training, HIIT may provide a stronger cardiopulmonary and peripheral metabolic stimulus within a shorter training time, while allowing intermittent recovery that may improve tolerability in selected patients (7, 8). Potential mechanisms through which HIIT may influence post-stroke function include improvements in aerobic capacity, peripheral oxygen utilization, lower-limb muscle performance, and neuromuscular recruitment. These adaptations may be particularly relevant to endurance-based walking capacity, such as 6MWT performance. However, improvements in aerobic or endurance capacity do not necessarily translate into better short-distance gait speed or gait control, because 10MWT performance also depends on task-specific motor control, gait symmetry, balance, coordination, and movement quality (9). Similarly, potential improvements in balance may reflect indirect effects on strength, postural adjustment capacity, and sensorimotor integration, but these mechanisms have not been directly tested in most HIIT trials and should therefore be interpreted cautiously. Feasibility and safety considerations for post-stroke HIIT have also been discussed in related feasibility studies and reviews (56, 62).

Several systematic reviews and meta-analyses have examined aerobic exercise, HIIT safety and feasibility, cardiorespiratory fitness, and mobility outcomes after stroke. However, important gaps remain. First, existing reviews have often focused on broad exercise categories or selected outcomes, rather than simultaneously examining balance, walking endurance, walking speed, cardiorespiratory fitness, blood pressure, and stroke severity within a single analytic framework. Second, post-stroke HIIT trials are typically small and heterogeneous with respect to intensity prescription, interval structure, training frequency, intervention duration, and comparator type. Third, individual trials often report multiple related outcomes or multiple effect sizes, which creates statistical dependence. When correlated effect sizes from the same study are treated as independent in a conventional two-level meta-analysis, standard errors may be biased and uncertainty may be estimated inefficiently. The issue is therefore not that conventional meta-analysis is inherently inappropriate, but that dependence among multiple effect sizes requires an analytic strategy that accounts for within-study correlation (10).

To address these issues, we conducted a systematic review and three-level random-effects meta-analysis of randomized controlled trials evaluating HIIT in post-stroke populations. The three-level model was used to account for sampling variance, within-study/effect-size-level variance, and between-study variance, and cluster-robust variance estimation with small-sample correction was used to support inference when multiple effect sizes were contributed by the same study. The primary aim was to estimate the effect of HIIT on balance after stroke. Secondary aims were to evaluate effects on walking endurance and short-distance walking speed or gait-control performance. Exploratory aims were to examine cardiorespiratory fitness, blood pressure, stroke severity, and whether study or training-load characteristics generated preliminary signals that may inform future hypothesis-driven trials.

## 2 Methods

### 2.1 Registration of systematic review protocol

This systematic review and meta-analysis was conducted in accordance with the PRISMA 2020 statement (11). The protocol was registered in PROSPERO (CRD42027809778).

### 2.2 Search strategy

We searched PubMed, Web of Science, Embase, Scopus, and the Cochrane Library from database inception to December 31, 2025. The final search was performed on December 31, 2025. Searches combined controlled vocabulary terms, where available, and free-text terms related to stroke and high-intensity interval training. Stroke-related terms included “stroke,” “cerebrovascular accident,” “ischemic stroke,” “ischaemic stroke,” “hemorrhagic stroke,” “haemorrhagic stroke,” and “CVA.” Intervention-related terms included “high-intensity interval training,” “HIIT,” “interval training,” “aerobic interval training,” “high-intensity training,” “high-intensity stepping,” and “speed-dependent treadmill training.” Randomized trial filters were also applied using terms such as “randomized,” “randomised,” “randomly,” “trial,” and “controlled trial.”

Search fields and syntax were adapted for each database. For example, PubMed searches used MeSH terms and title/abstract fields; Embase searches used Emtree terms and title/abstract/keyword fields; Web of Science and Scopus searches used topic or title/abstract/keyword fields; and Cochrane Library searches used title, abstract, and keyword fields. The complete database-specific search strategies, including Boolean operators, search fields, and limits, are provided in Supplementary material.

Only English-language full-text publications were included. This restriction was applied because resources for translation and verification of non-English trial reports were not available. We acknowledge that this may have introduced language bias, and this limitation was considered when interpreting the certainty and generalizability of the evidence. Reference lists of included studies and relevant reviews were manually screened to identify additional eligible studies. Trial registries, gray literature databases, preprint servers, and conference proceedings were not systematically searched.

### 2.3 Study selection and eligibility criteria

All records were imported into NoteExpress version 3.2.0 for de-duplication. Two reviewers independently screened titles and abstracts and then assessed the full texts of potentially eligible studies. Disagreements were resolved through discussion, and a third reviewer adjudicated when consensus could not be reached. The study selection process was documented using a PRISMA flow diagram.

Studies were eligible if they met the following criteria: (1) randomized controlled trials published in English; (2) adult participants with clinically diagnosed stroke; (3) an intervention that included HIIT as the primary exercise component or as the main distinguishable high-intensity interval component; (4) a comparator consisting of usual care, conventional rehabilitation, moderate-intensity continuous training, low-intensity interval training, lower-intensity exercise, or no additional exercise intervention; and (5) at least one eligible functional, physiological, or clinical outcome relevant to post-stroke rehabilitation.

HIIT was operationally defined as repeated bouts of relatively high-intensity exercise separated by active or passive recovery periods. Eligible interventions had to report at least one of the following components: intensity target or monitoring method, such as percentage of peak heart rate, maximum heart rate, heart-rate reserve, oxygen uptake, rating of perceived exertion, treadmill speed, stepping intensity, or another stated intensity metric; interval or bout structure; recovery structure; session duration; and training frequency or intervention duration. Interventions such as HIIT alone, HIIT combined with moderate-intensity continuous training, speed-dependent treadmill training, and high-intensity stepping training were eligible when the high-intensity interval component could be identified and the intervention effect could be extracted or reasonably attributed to the HIIT-based program.

Balance assessed using the Berg Balance Scale was defined as the primary outcome of this review. Walking endurance, measured using the 6-min walk test, and short-distance walking speed or gait-control performance, measured using the 10-meter walk test, were defined as key secondary functional outcomes. Cardiorespiratory fitness, systolic blood pressure, diastolic blood pressure, and stroke severity, including the Scandinavian Stroke Scale, were treated as exploratory physiological or clinical outcomes. Individual trials were not required to designate balance as their own primary endpoint; rather, trials were eligible if they reported at least one of the eligible functional, physiological, or clinical outcomes listed above. Studies were excluded if they were non-randomized studies, duplicate reports, conference abstracts without full data, narrative reviews, protocols without results, or studies without extractable quantitative data. Studies were also excluded if the intervention was a multicomponent exercise program in which the independent contribution of the HIIT-based component could not be isolated, or if outcome data could not be transformed into means and standard deviations and could not be obtained from the authors.

### 2.4 Data extraction

Two reviewers independently extracted data using a pre-designed Microsoft Excel spreadsheet. Extracted information included study characteristics, participant characteristics, intervention characteristics, comparator characteristics, outcomes, and methodological information required for risk-of-bias assessment. Study-level information included first author, publication year, country or region, study design, sample size, and intervention setting. Participant-level information included age, sex, body mass index when reported, stroke type, stroke stage or time since stroke, baseline severity, and relevant eligibility criteria. Intervention-level information included exercise modality, intensity target or monitoring method, bout duration, recovery duration, number of intervals, work-to-recovery structure, total session duration, training frequency, intervention duration, adherence, and adverse-event reporting. Comparator information included usual care, conventional rehabilitation, lower-intensity exercise, moderate-intensity continuous training, low-intensity interval training, sham intervention, or no additional intervention. For each eligible outcome, means, standard deviations, sample sizes, and time points were extracted where available. When change scores and change-score standard deviations were reported, these values were used preferentially. When only baseline and post-intervention values were reported, change-score standard deviations were derived using an assumed pre-post correlation, as described below. When outcome data were presented graphically, numerical values were extracted using WebPlotDigitizer version 4.1 (60). Studies were excluded from quantitative synthesis only when key numerical data remained unavailable after reasonable attempts to extract or obtain them (11).

### 2.5 Effect size calculation

Effect sizes were calculated as standardized mean differences and expressed as Hedges' g to account for small-sample bias. The primary effect-size metric was based on the between-group difference in change scores.

For each study, the change score in the HIIT group and control group was calculated as:


$$\begin{array}{l} {\text{Δ}}_{\text{T}}={\text{M}}_{\text{post},\text{T}}-{\text{M}}_{\text{pre},\text{T}}\\ {\text{Δ}}_{\text{C}}\;={\text{ M}}_{\text{post},\text{C}}-{\text{M}}_{\text{pre},\text{C}} \end{array}$$

where T denotes the HIIT or intervention group and C denotes the control group. The uncorrected standardized mean difference was calculated as:


$$\text{d = }\frac{{\text{Δ}}_{\text{T}}-{\text{Δ}}_{\text{C}}}{{\text{SD}}_{\text{pooled},\text{Δ}}}$$

where SD_pooled, Δ_ is the pooled standard deviation of the change scores:


$$\begin{array}{l} \text{S}{\text{D}}_{\text{pooled},\text{Δ }}=\;\sqrt{\frac{\left({\text{n}}_{\text{T}}-1\right)\text{S}{\text{D}}_{\text{Δ},\text{T}}^{2}+\left({\text{n}}_{\text{C}}-1\right)\text{S}{\text{D}}_{\text{Δ},\text{C}}^{2}}{{\text{n}}_{\text{T }}+{\text{n}}_{\text{C}}-2}} \end{array}$$

Hedges' g was then calculated using the small-sample correction factor:


$$\begin{array}{l} \text{g }=\text{ J }\times \text{ d}\\ \text{J}=\text{ 1 }-\;\frac{\text{3}}{\text{4}\left({\text{n}}_{\text{T}}\text{+ }{\text{n}}_{\text{C}}-2\right)-1} \end{array}$$

The sampling variance for each standardized mean difference was estimated using the standard large-sample approximation for Hedges' g:


$$\begin{array}{l} {\text{V}}_{\text{g}}\;={\text{ J}}^{2}\left(\frac{{\text{n}}_{\text{T}}\;+\;{\text{n}}_{\text{C}}}{{\text{n}}_{\text{T}}{\text{n}}_{\text{C}}}\;+\;\frac{{\text{d}}^{2}}{2\left({\text{n}}_{\text{T}}\;+\;{\text{n}}_{\text{C}}-2\right)}\right) \end{array}$$

When change-score standard deviations were not reported, they were derived from baseline and post-intervention standard deviations using the following formula:


$$\begin{array}{l} \text{S}{\text{D}}_{\text{Δ}}=\sqrt{\text{S}{\text{D}}_{\text{pre}}^{2}\;+\text{ S}{\text{D}}_{\text{post}}^{2}\;-\;2\text{r}\left(\text{S}{\text{D}}_{\text{pre}}\right)\;\left(\text{S}{\text{D}}_{\text{post}}\right)} \end{array}$$

The primary analysis assumed a pre-post correlation of *r* = 0.5. The number of effect sizes requiring imputed change-score standard deviations, the outcomes affected, and the influence of alternative assumed correlations are reported in Supplementary material. All effect sizes were coded so that positive values indicated a greater favorable change in the HIIT group compared with the control group. For outcomes where lower values indicate improvement, including 10-m walk test time and blood pressure indices, the direction of effect was reversed before pooling so that positive effect sizes consistently favored HIIT.

### 2.6 Risk of bias assessment

Risk of bias in the included randomized controlled trials was assessed using the Cochrane Risk of Bias 2 tool. RoB 2 evaluates five domains: bias arising from the randomization process, bias due to deviations from intended interventions, bias due to missing outcome data, bias in measurement of the outcome, and bias in selection of the reported result. Two reviewers independently completed the assessments and cross-checked judgments. Disagreements were resolved by discussion or consultation with a third reviewer. Each domain and the overall risk of bias were judged as “low risk,” “some concerns,” or “high risk.”

### 2.7 Statistical analysis

All analyses were conducted in *R* using the metafor and clubSandwich packages. Because individual trials could contribute multiple statistically dependent effect sizes, such as multiple outcomes, multiple intervention arms, shared control groups, or multiple time points, we used three-level random-effects models. The models partitioned variability into sampling variance at level 1, within-study or effect-size-level variance at level 2, and between-study variance at level 3.

The general model structure was:


$$\begin{array}{l} {\text{y}}_{\text{ij}}=\text{μ }+\;{\text{u}}_{\text{j }}+\;{\text{v}}_{\text{ij }}+\;{\text{e}}_{\text{ij}} \end{array}$$

where y_ij_ is the *i*th effect size from study j, μ is the pooled mean effect, u_j_ is the between-study random effect, v_ij_ is the within-study random effect, and e_ij_ is sampling error. Variance components were reported as τ^2^ at the within-study and between-study levels.

Study ID was used as the clustering unit for cluster-robust variance estimation. Statistical inference for pooled effects, including standard errors, 95% confidence intervals, and *p*-values, was based on CR2 cluster-robust variance estimation with Satterthwaite small-sample degrees-of-freedom correction. This approach was used to reduce the risk of inflated precision when multiple effect sizes originated from the same trial. When multiple intervention arms shared a control group, the dependency was handled within the multilevel structure and through clustering at the study level. When multiple time points were eligible and extracted, they were treated as dependent effect sizes nested within the original study. Heterogeneity was summarized using three-level variance components. The conventional *I*^2^ statistic from two-level meta-analysis was not used as the primary heterogeneity measure because *I*^2^ is not uniquely defined in the same way for three-level models. Statistical significance was evaluated using two-sided tests with *p* < 0.05.

Meta-regression and subgroup analyses were conducted only as exploratory, hypothesis-generating analyses. These analyses were not designed to establish causal moderator effects or optimal HIIT prescriptions. Candidate moderators included stroke stage or severity, publication region, intervention duration, training frequency, session duration, weekly training volume, comparator type, and adverse-event reporting, when sufficient data were available. Subgroup thresholds were selected according to the distribution of intervention characteristics across included studies and clinical interpretability. These thresholds included training frequency of ≤ 3 vs. ≥4 sessions per week, intervention duration of ≤ 4 vs. ≥5 weeks, session duration of < 30 vs. ≥30 min, and weekly training volume of < 600 vs. ≥600 MET·min/week. These thresholds were not pre-specified in the PROSPERO protocol and should therefore be interpreted as *post hoc* exploratory classifications. No multiplicity adjustment was applied; therefore, subgroup and meta-regression results were interpreted cautiously. Publication bias and small-study effects were assessed using contour-enhanced funnel plots and Egger's regression test when sufficient outcome-specific data were available. Because several outcomes included a small number of studies or effect sizes, publication-bias assessments were considered underpowered. Therefore, non-significant Egger's tests were not interpreted as evidence that publication bias was absent.

### 2.8 Certainty assessment

The certainty of evidence for each outcome was assessed using the Grading of Recommendations Assessment, Development and Evaluation approach. Because all included studies were randomized controlled trials, the initial certainty rating was high and was downgraded when concerns were identified. The domains considered were risk of bias, inconsistency, indirectness, imprecision, and publication bias. For each outcome, the GRADE evidence profile included the number of studies, number of participants, pooled effect estimate with 95% confidence interval, certainty rating, and detailed reasons for downgrading. Certainty was categorized as high, moderate, low, or very low. These judgments were used to guide the interpretation of clinical implications and to ensure that conclusions reflected the strength and limitations of the available evidence.

## 3 Results

### 3.1 Search results

**Figure 1:**
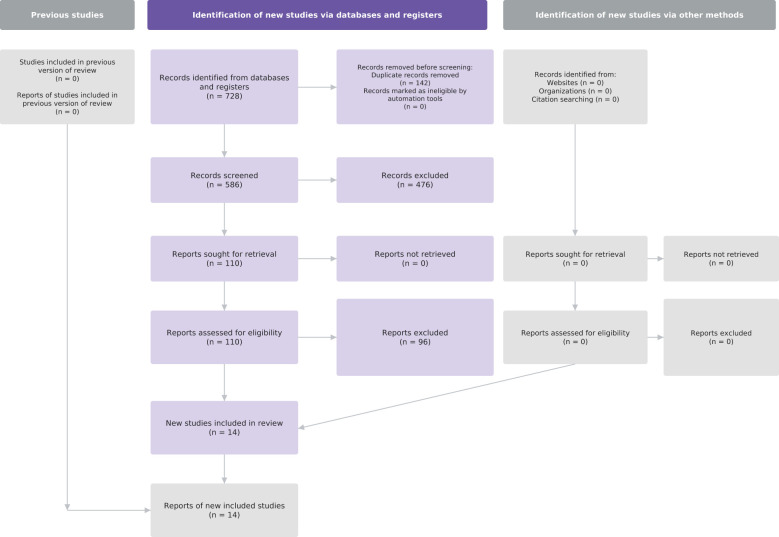
PRISMA flow diagram detailing the study inclusion process.

Of the 728 records initially identified, 142 duplicates were removed using NoteExpress (version 3.2.0). The remaining 586 records were screened by title and abstract, after which 110 articles were assessed for full-text eligibility. Following full-text evaluation, 13 English-language randomized controlled trials were included in the final quantitative synthesis. The numbers reported in the text were checked against the PRISMA flow diagram to ensure consistency. The study selection process is presented in Figure 1, which has been replaced with a higher-resolution version to improve readability.

### 3.2 Study characteristics

Fourteen randomized controlled trials involving 717 post-stroke participants were included. In the intervention arms, participants received HIIT or HIIT-based interventions, including HIIT alone, HIIT combined with moderate-intensity continuous training, speed-dependent treadmill training, or high-intensity stepping training when the high-intensity interval component could be identified. Control conditions consisted of usual rehabilitation care, conventional rehabilitation, lower-intensity exercise training, moderate-intensity continuous training, low-intensity interval training, sham intervention, or no additional exercise intervention. Intervention duration ranged from 3 to 24 weeks across studies. Only one trial directly compared HIIT with low-intensity interval training. Balance assessed using the Berg Balance Scale was defined as the primary outcome of this review. Walking endurance, measured using the 6-min walk test, and short-distance walking speed or gait-control performance, measured using the 10-meter walk test, were considered key secondary functional outcomes. Cardiorespiratory fitness, stroke severity, and blood pressure indices were treated as exploratory physiological or clinical outcomes. Participant characteristics, intervention characteristics, comparator conditions, and safety information are presented in Tables 1, 2.

**Table 1:** Basic characteristics of the included studies.

Author	Year	Study design	Country	BMI	Age	Sex (M/W)	Condition	*n*	Intervention group	*n*	Control group	Outcomes
Amanzonwé et al. (42)	2025	RCT	Benin	*T* = 25.5 ± 1.8; *C* = 25.4 ± 1.2	T: 56.6 ± 12.9; *C*: 56.9 ± 13.0	M: 24; W: 20	Stroke patients	22	HIIT + SHAM	22	SHAM	①②③
Gjellesvik et al. (43)	2021	RCT	Norway	*T* = 26.9 ± 3.7; *C* = 27.8 ± 4.8	*T*: 57.6 ± 9.2; *C*: 58.7 ± 9.2	M: 29; W: 41	Stroke patients	36	HIIT	34	CON	①②③
Globas et al. (44)	2012	RCT	Switzerland	*T* = 25.9 ± 3.4; *C* = 26.8 ± 3.4	*T*: 68.6 ± 6.7; *C*: 68.7 ± 6.1	M: 29; W: 7	Stroke patients	18	HIIT	18	CON	①②③④
Hornby et al. (45)	2022	RCT	USA	N/A	*T*: 52 ± 13; *C*: 60 ± 9.2	M: 32; W: 12	Stroke patients	27	HIIT	17	CON	⑦
Krawcyk et al. (46)	2023	RCT	Denmark	*T* = 28 ± 5; *C* = 25 ± 4	*T*: 64.4 ± 8.5; *C*: 63.4 ± 9.2	M: 48; W: 11	Stroke patients	28	HIIT	31	CON	⑤⑥
Lamberti et al. (47)	2017	RCT	Italy	*T* = 26 ± 4; *C* = 27 ± 5	*T*: 67 ± 10; *C*: 69 ± 9	M: 27; W: 8	Stroke patients	17	HIIT	18	LIIT	①②③
Lapointe et al. (48)	2023	RCT	Canada	*T* = 27.9 ± 2.4; *C* = 28.4 ± 5.4	*T*: 71.8 ± 9.9; *C*: 65.6 ± 11.3	M: 23; W: 12	Stroke patients	19	HIIT + MICT	16	MICT	④⑤⑥
Lau et al. (49)	2011	RCT	China	N/A	*T*: 69.5 ± 11.1; *C*: 72.1 ± 9.2	M: 21; W: 9	Stroke patients	15	HIIT	15	CON	①
Moore et al. (50)	2020	RCT	Norway	N/A	*T*: 73 ± 10; *C*: 74 ± 14	M: 64; W: 47	Stroke patients	54	HIIT	56	CON	①②⑦
Pang et al. (51)	2005	RCT	Italy	N/A	*T*: 65.8 ± 9.1; *C*: 64.7 ± 8.4	M: 37; W: 26	Stroke patients	32	HIIT	31	CON	①②④
Levy et al. (52)	2015	RCT	Spain	N/A	*T*: 64.1 ± 12.3; *C*: 58.9 ± 14.6	M: 17; W: 21	Stroke patients	19	HIIT	19	CON	①④
Sandberg et al. (53)	2016	RCT	Sweden	N/A	*T*: 71.3 ± 7.0; *C*: 70.4 ± 8.1	M: 28; W: 28	Stroke patients	29	HIIT	27	CON	①②③
Krawcyk et al. (54)	2019	RCT	Denmark	*T* = 27.5 ± 4.5; *C* = 25.6 ± 3.6	*T*: 63.7 ± 8.9; *C*: 63.7 ± 9.2	M: 49; W: 14	Stroke patients	31	HIIT	32	CON	⑤⑥
Zaman et al. (55)	2025	RCT	Pakistan	N/A	*T*: 48.2 ± 4.9; *C*: 50.5 ± 4.3	M: 18; W: 16	Stroke patients	17	HIIT	17	CON	①②③

T: experimental group; C: control group; ① BBS: Balance Scale; ② 6MWT: 6-min walk test; ③ 10MWT: 10-meter walk test; ④ CRF: Cardiorespiratory fitness; ⑤ DBP: Diastolic blood pressure; ⑥ SBP: Systolic blood pressure; ⑦ SSS: Stroke Scale.

**Table 2:** Experimental design of included literature.

Author	Condition status	Group allocation	Warm-up	Bout duration	Exercise intensity	Recovery	No. of intervals	Total session duration	Training frequency	Training period	Adverse events
Amanzonwé et al. (42)	Early-stage stroke patients	HIIT + SHAM/SHAM	1 × 3 min (N/A)	1 min	70%−80% HRpeak	4 min (N/A)	4–6	20–30 min	Three sessions/week	6 weeks	Not reported
Gjellesvik et al. (43)	3 months to 5 years post-stroke	HIIT/CON	1 × 10 min	4 min	85%−95% HRpeak	3 min (N/A)	1	17 min	Three sessions/week	8 weeks	Not reported
Globas et al. (44)	6 months post-stroke	HIIT/CON	N/A	N/A	60%−80% HRpeak	N/A	N/A	30–50 min	Three sessions/week	8 weeks	Two participants had transportation issues; one participant had a hip fracture
Hornby et al. (45)	1 to 6 months post-stroke	HIIT/CON	N/A	N/A	70%−80% HRpeak	N/A	N/A	40 min	Four sessions/week	4 weeks	Not reported
Krawcyk et al. (46)	6 to 12 months post-stroke	HIIT/CON	N/A	N/A	N/A	N/A	N/A	15 min	Five sessions/week	12 weeks	Not reported
Lamberti et al. (47)	Chronic stroke patients	HIIT/LIIT	N/A	5–10 min	60%−70% HRpeak (work); 50% HRpeak (recovery)	20–30 s (N/A)	8	30 min	Three sessions/week	8 weeks	Not reported
Lapointe et al. (48)	3 months post-stroke	HIIT + MICT/MICT	1 × 5 min + 1 × 5 min	N/A	95% HRpeak (RPE 4–6)	1 min (N/A)	N/A	20–40 min	Three sessions/week	24 weeks	Not reported
Lau et al. (49)	1 month post-stroke	HIIT/CON	N/A	30 s	Maximum safe speed	2 min (N/A)	7–8	30 min	Three sessions/week	4 weeks	Two participants refused to continue outpatient training; one recurrent stroke; one acute cardiac disease
Moore et al. (50)	2 months post-stroke	HIIT/CON	N/A	N/A	70%−85% HRR	N/A	N/A	45–60 min	Four sessions/week	3 weeks	Not reported
Pang et al. (51)	1 year post-stroke	HIIT/CON	N/A	N/A	70%−80% HRR	1 min (N/A)	N/A	30 min	Three sessions/week	19 weeks	One participant felt exercise was too tiring; one had no time; one lost to follow-up; one could not commit time
Levy et al. (52)	6 months post-stroke	HIIT/CON	1 × 5 min	1 min	85% HRpeak	5 min (N/A)	N/A	45 min	Three sessions/week	8 weeks	Not reported
Sandberg et al. (53)	Early-stage stroke patients	HIIT/CON	N/A	8 min	50%−80% HRpeak	N/A	2	30 min	Two sessions/week	8 weeks	One recurrent stroke; one unknown reason
Krawcyk et al. (54)	3 weeks post-stroke	HIIT/CON	N/A	3 min	77%−93% HRpeak	2 min (N/A)	3	15 min	Five sessions/week	12 weeks	One acute low back pain; one nausea/dizziness/stomach pain; one bilateral knee osteoarthritis; one affected-limb fatigue; one technical issue
Zaman et al. (55)	3 months to 5 years post-stroke	HIIT/CON	1 × 3 min	3 min	60% HRpeak	2 min (N/A)	4	45–50 min	Five sessions/week	4 weeks	Two participants had transportation issues; one recurrent stroke; one death

### 3.3 Risk of bias and quality assessment

**Figure 2:**
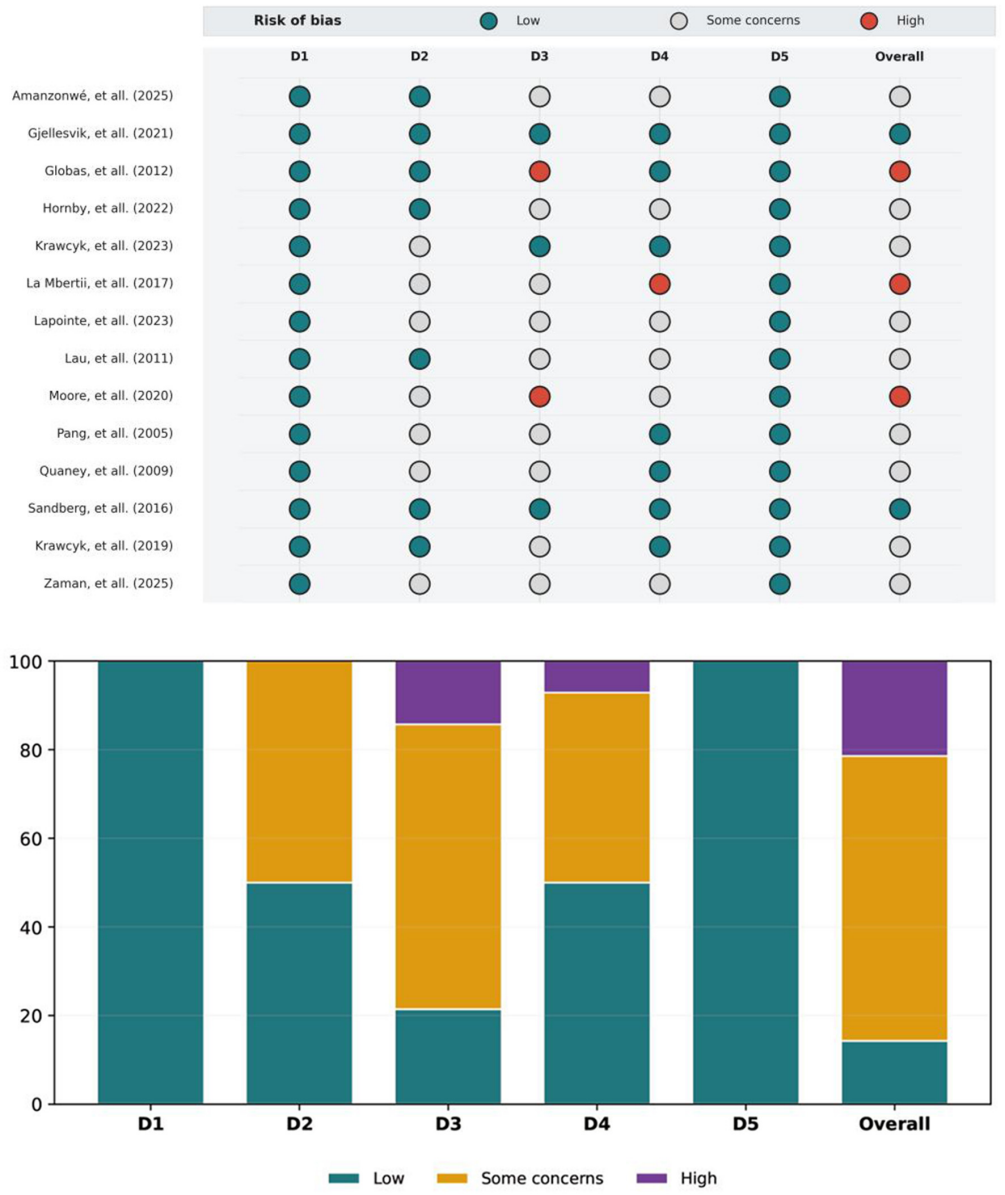
Risk of bias assessment for all included studies.

Using the Cochrane Risk of Bias 2 tool, we assessed risk of bias for the 14 included trials. Most studies were rated as having some concerns (9/14, 64.3%), followed by high risk (3/14, 21.4%); only two studies (2/14, 14.3%) were judged as low risk overall. Therefore, most of the available evidence was derived from trials with either some methodological concerns or high risk of bias. By domain, the randomization process (D1) and bias in selection of the reported result (D5) were rated as low risk in all studies (14/14, 100%). For deviations from intended interventions (D2), low risk and some concerns each accounted for 50.0% of studies (7/14 vs. 7/14). Concerns were most prominent for missing outcome data (D3), with some concerns in 10/14 studies (71.4%) and high risk in 2/14 studies (14.3%). For measurement of the outcome (D4), 7/14 studies (50.0%) were rated as low risk, 6/14 studies (42.9%) as having some concerns, and 1/14 study (7.1%) as high risk. Overall, uncertainty in study quality was driven primarily by missing outcome data and, to a lesser extent, deviations from intended interventions and outcome measurement. These limitations were incorporated into the interpretation of pooled effects and the GRADE certainty assessment. In particular, statistically marginal findings and findings based on sparse outcome-specific data should be interpreted cautiously. The risk of bias assessment is presented in Figure 2.

### 3.4 Main effect

**Figure 3:**
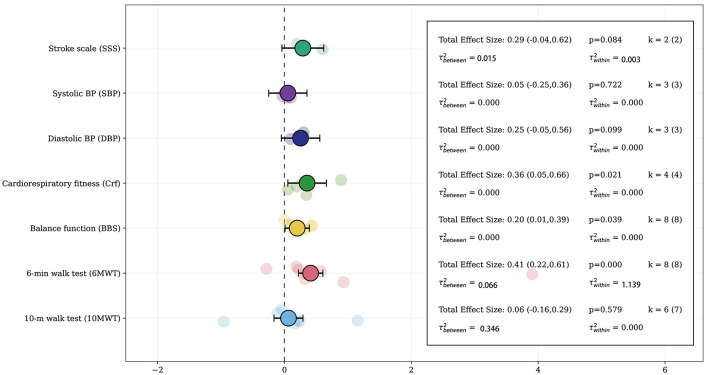
Meta-analysis of the influence of effect of HIIT on related indicators of stroke patients.

Pooled effect sizes (ES; Hedges' g), 95% confidence intervals, and *p*-values were derived from three-level random-effects models with cluster-robust variance estimation and small-sample correction. Positive effect sizes were coded to favor HIIT across all outcomes. For outcomes in which lower values indicate better performance, including 10MWT time and blood pressure indices, effect directions were harmonized before pooling so that positive values consistently indicated greater favorable change in the HIIT group. Heterogeneity was summarized using variance components at the between-study level and the within-study/effect-size level. The conventional *I*^2^ statistic from two-level models was not used as the primary heterogeneity metric. For balance, eight effect sizes from seven studies were included for the Berg Balance Scale. The pooled ES was 0.20 (95% CI 0.01 to 0.39, *p* = 0.039), with τ^2^_between ≈ 0 and τ^2^_within ≈ 0. This result suggested a small favorable effect of HIIT on balance; however, the lower bound of the confidence interval was close to the null value, and the certainty of evidence was low. For walking endurance, eight effect sizes were included for the 6-min walk test. The pooled ES was 0.41 (95% CI 0.22 to 0.61, *p* < 0.001), with τ^2^_between = 0.066 and τ^2^_within = 1.139. Although the average effect was statistically significant, the large within-study/effect-size variance component indicated heterogeneity in the magnitude of the effect across dependent effect sizes and study conditions. Therefore, this finding should be interpreted as a favorable average effect rather than evidence of a uniform response across all HIIT protocols or post-stroke populations. For short-distance walking speed or gait-control performance, seven effect sizes from six studies were included for the 10-meter walk test. After harmonizing effect direction, the pooled ES was 0.06 (95% CI−0.16 to 0.29, *p* = 0.579), with τ^2^_between = 0.346 and τ^2^_within ≈ 0. The pooled estimate was close to zero and the confidence interval crossed the null value, indicating that current evidence does not demonstrate a clear effect of HIIT on 10MWT performance. For cardiorespiratory fitness, four effect sizes were included and the pooled ES was 0.36 (95% CI 0.05 to 0.66, *p* = 0.021), with τ^2^_between ≈ 0 and τ^2^_within ≈ 0. This finding suggested a possible favorable effect of HIIT on cardiorespiratory fitness, although the certainty of evidence was very low and the result should be interpreted cautiously. For blood pressure, SBP and DBP each included three effect sizes. The pooled ES was 0.05 (95% CI−0.25 to 0.36, *p* = 0.722) for SBP and 0.25 (95% CI−0.05 to 0.56, *p* = 0.099) for DBP, with variance components close to zero at both levels. Neither effect reached statistical significance. For stroke severity, assessed using the Scandinavian Stroke Scale, two effect sizes were included. The pooled ES was 0.29 (95% CI−0.04 to 0.62, *p* = 0.084), with τ^2^_between = 0.015 and τ^2^_within = 0.003. Interpretation of this result is limited by sparse data and imprecision. Taken together, pooled estimates suggested possible favorable effects of HIIT on BBS, 6MWT, and cardiorespiratory fitness, whereas effects on 10MWT, SBP, DBP, and SSS were uncertain. These results should be interpreted in the context of risk of bias, heterogeneity, limited outcome-specific evidence, and low to very low certainty of evidence. The pooled effects across outcomes are presented in Figure 3.

### 3.5 Meta-regression and subgroup analyses of moderators

Meta-regression was performed for the 6MWT and 10MWT outcomes (Tables 3, 4). Six candidate moderators were selected based on clinical relevance and the available study characteristics: stroke severity or stage, publication region, intervention duration, training frequency, session duration, and adverse-event reporting. The meta-regression analysis for 6MWT showed that publication region was statistically associated with effect size (*p* = 0.042), whereas the other moderators showed *p*-values greater than 0.05. This finding should be interpreted only as a preliminary signal, because publication region may be confounded by rehabilitation setting, intervention modality, comparator type, baseline severity, sample size, and publication year. For 10MWT, no clear moderator association was identified.

**Table 3:** Meta-regression analysis of different moderating variables on 6MWT in post-stroke patients.

Moderators	β regression coefficient	Standard error	*t*-value	*P* > |*t*|	95% CI
Stroke severity	−0.629	0.368	−1.708	0.138	[−1.530, 0.272]
Publication region	−0.685	0.303	−2.572	0.042	[−3.456, −0.086]
Intervention duration	−0.075	0.089	−0.843	0.432	[−0.293, 0.143]
Training frequency	0.889	0.434	2.049	0.086	[−0.172, 1.951]
Session duration	0.031	0.038	0.828	0.439	[−0.061, 0.123]
Adverse events	0.716	0.819	0.874	0.416	[−1.289, 2.721]

**Table 4:** Meta-regression analysis of different moderating variables on 10MWT in post-stroke patients.

Moderators	β regression coefficient	Standard error	*t*-value	*p* > |*t*|	95% CI
Stroke severity	−0.053	0.215	−0.246	0.818	[−0.651, 0.545]
Publication region	−0.104	0.476	−0.217	0.839	[−1.426, 1.219]
Intervention duration	−0.075	0.146	−0.407	0.705	[−0.862, 0.180]
Training frequency	−0.341	0.188	−1.818	0.143	[−0.172, 1.951]
Session duration	−0.020	0.020	−1.003	0.372	[−0.076, 0.036]
Adverse events	−0.205	0.435	−0.471	0.662	[−1.411, 1.002]

Due to the relatively small number of effect sizes for other outcomes, subgroup analyses were conducted only for BBS, 6MWT, and 10MWT. The subgroup results are presented in Table 5. For BBS, numerically larger effects were observed in some subgroups, including interventions with lower weekly frequency, longer intervention duration, shorter session duration, and lower weekly training volume. For 6MWT, numerically larger effects were observed in some subgroups characterized by higher weekly frequency, longer intervention duration, shorter session duration, and higher weekly training volume. For 10MWT, subgroup estimates were inconsistent and did not support a clear conclusion regarding the effect of HIIT on short-distance walking speed or gait-control performance.

**Table 5:** Subgroup analysis of the subgroup effects of different moderating variables on post-stroke patients.

Moderator	Subgroup	BBS effect size and 95% CI	6MWT effect size and 95% CI	10MWT effect size and 95% CI
Stroke severity	≤ 2 months	0.10 (−0.12, 0.35)	0.51 (0.21, 0.97)	−0.01 (−1.02, 1.09)
	≥3 months	0.35 (0.16, 0.81)^*^	0.76 (0.13, 1.67)^*^	−0.11 (−0.38, 0.25)
Publication region	Europe	0.25 (0.11, 0.73)^*^	0.35 (0.26, −0.84)^*^	0.10 (−0.38, 0.45)
	Asia	0.14 (−0.23, 0.32)	−0.87 (−1.21, −0.25)^*^	−0.68 (−1.32, −0.05)^*^
	Africa	0.16 (−0.26, 0.34)	−0.53 (−1.06, 0.01)	0.38 (−0.70, 0.46)
Training dose	< 30 min	0.69 (0.45, 0.93)^*^	−0.62 (−0.89, −0.36)^*^	−0.64 (−1.58, −0.04)^*^
	≥30 min	0.18 (−0.14, 0.50)	−0.39 (−0.68, −0.09)^*^	0.16 (−0.29, 0.11)
Total training volume	< 600 MET·min/week	0.51 (0.31, 0.97)^*^	0.57 (0.23, 0.90)^*^	−0.24 (−0.35, −0.04)^*^
	≥600 MET·min/week	0.03 (−0.26, 0.35)	0.72 (0.31, 1.56)^*^	0.06 (−0.38, 0.25)
Training frequency	≤ 3 times/week	0.39 (0.12, 0.73)^*^	0.36 (0.13, 0.97)^*^	−0.14 (−0.25, 0.04)
	≥4 times/week	0.15 (−0.15, 1.24)	0.97 (0.37, 1.54)^*^	0.11 (−0.15, 0.24)
Training duration	≤ 4 weeks	0.18 (0.11, 0.56)	0.22 (−0.53, 0.68)	0.11 (−0.16, 0.28)
	≥5 weeks	0.34 (−0.15, 1.24)^*^	0.86 (0.37, 1.54)^*^	−0.12 (−0.67, 0.43)
Control intensity	Placebo group	0.44 (0.27, 0.79)^*^	0.93 (0.52, 1.48)^*^	−1.55 (−1.78, −0.54)^*^
	Low dose group	0.04 (−0.29, 0.38)	−0.37 (−0.77, 0.28)	0.78 (0.38, 1.04)^*^
	Conventional group	0.41 (0.21, 0.68)^*^	0.59 (0.38, 1.14)^*^	−0.16(−0.29, 0.31)

^*^Indicates statistical significance at *p* < 0.05.

These subgroup patterns should not be interpreted as evidence that any specific combination of training frequency, intervention duration, session duration, or weekly training volume is optimal. Rather, they represent preliminary descriptive signals that may inform hypotheses for future dose-finding trials. Comparisons involving low-intensity interval training, sham controls, and region-specific effects were based on limited data and may be influenced by differences in intervention design, control conditions, adherence, achieved intensity, baseline function, and reporting quality.

### 3.6 Sensitivity analysis

**Figure 4:**
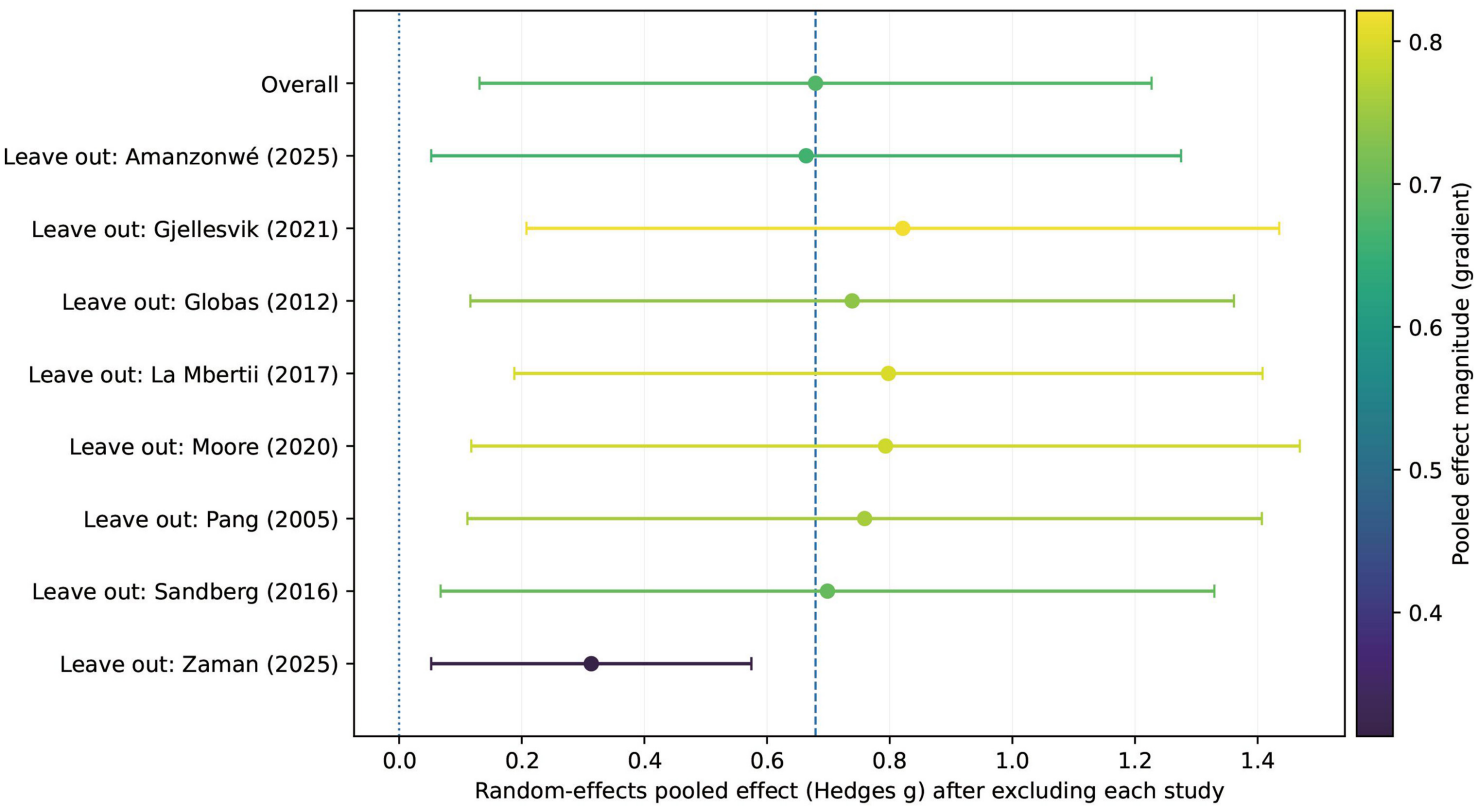
Leave-one-out sensitivity analysis of high-intensity interval training on 6MWT.

**Figure 5:**
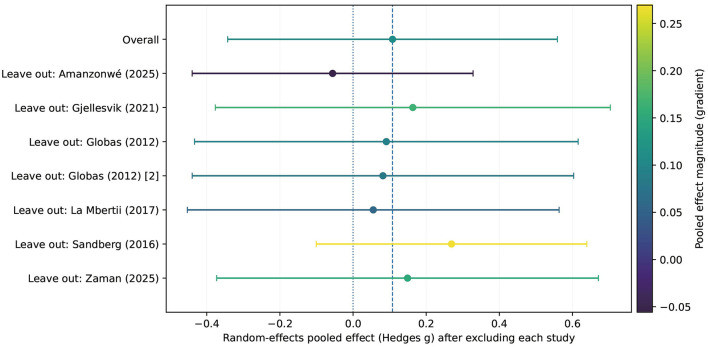
Sensitivity analysis of high-intensity interval training on 10MWT.

Sensitivity analyses were performed to assess whether the pooled effects were disproportionately influenced by individual studies (Figures 4, 5). Leave-one-out analyses were conducted for outcomes with sufficient effect sizes, particularly 6MWT and 10MWT. For 6MWT, the pooled average effect remained statistically favorable when individual studies were removed one at a time, suggesting that the statistical significance of the average effect was not driven by a single study. However, this result does not eliminate heterogeneity. The relatively large within-study/effect-size variance component for 6MWT indicates that the magnitude of the effect varied across study conditions and dependent effect sizes. For 10MWT, leave-one-out analyses indicated less stable results. The pooled estimate remained small and statistically uncertain, and the confidence interval continued to include the null value. These findings support a cautious interpretation that current evidence does not demonstrate a clear effect of HIIT on short-distance walking speed or gait-control performance. Because only two of the 14 included trials were judged as low risk overall and three studies were judged as high risk, sensitivity analyses excluding high-risk studies were considered. For outcomes with sufficient remaining studies after excluding high-risk trials, the direction and interpretation of the pooled estimates were examined. For outcomes with too few remaining studies or effect sizes, this analysis was not considered statistically reliable. Overall, the sensitivity analyses supported cautious interpretation of the findings, particularly for outcomes with marginal statistical significance, sparse evidence, or substantial heterogeneity.

### 3.7 Publication bias assessment

**Figure 6:**
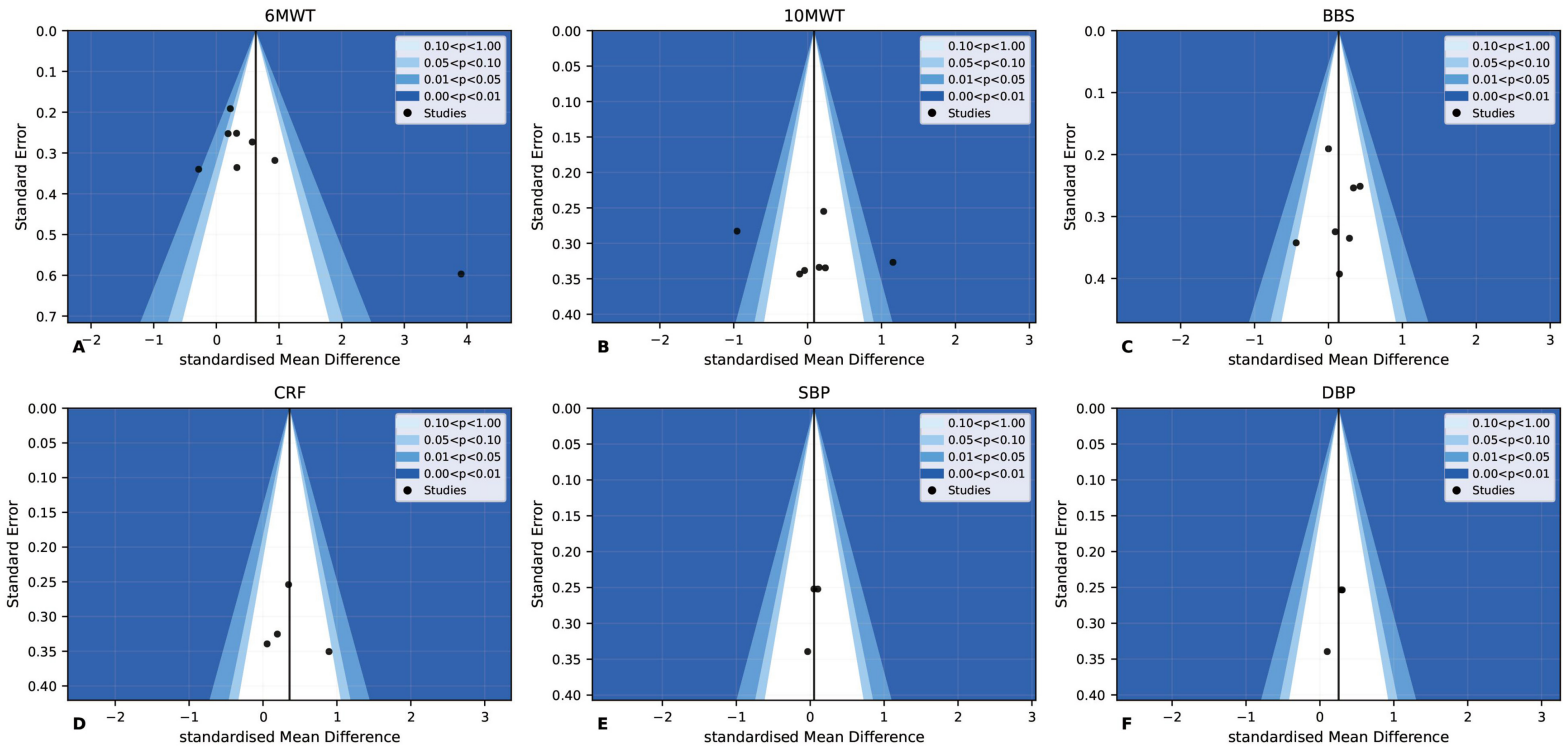
Funnel plots for publication-bias assessment: **(A)** 6-min walk test (6MWT); **(B)** 10-meter walk test (10MWT); **(C)** Berg Balance Scale (BBS); **(D)** cardiorespiratory fitness (CRF); **(E)** systolic blood pressure (SBP); and **(F)** diastolic blood pressure (DBP).

**Figure 7:**
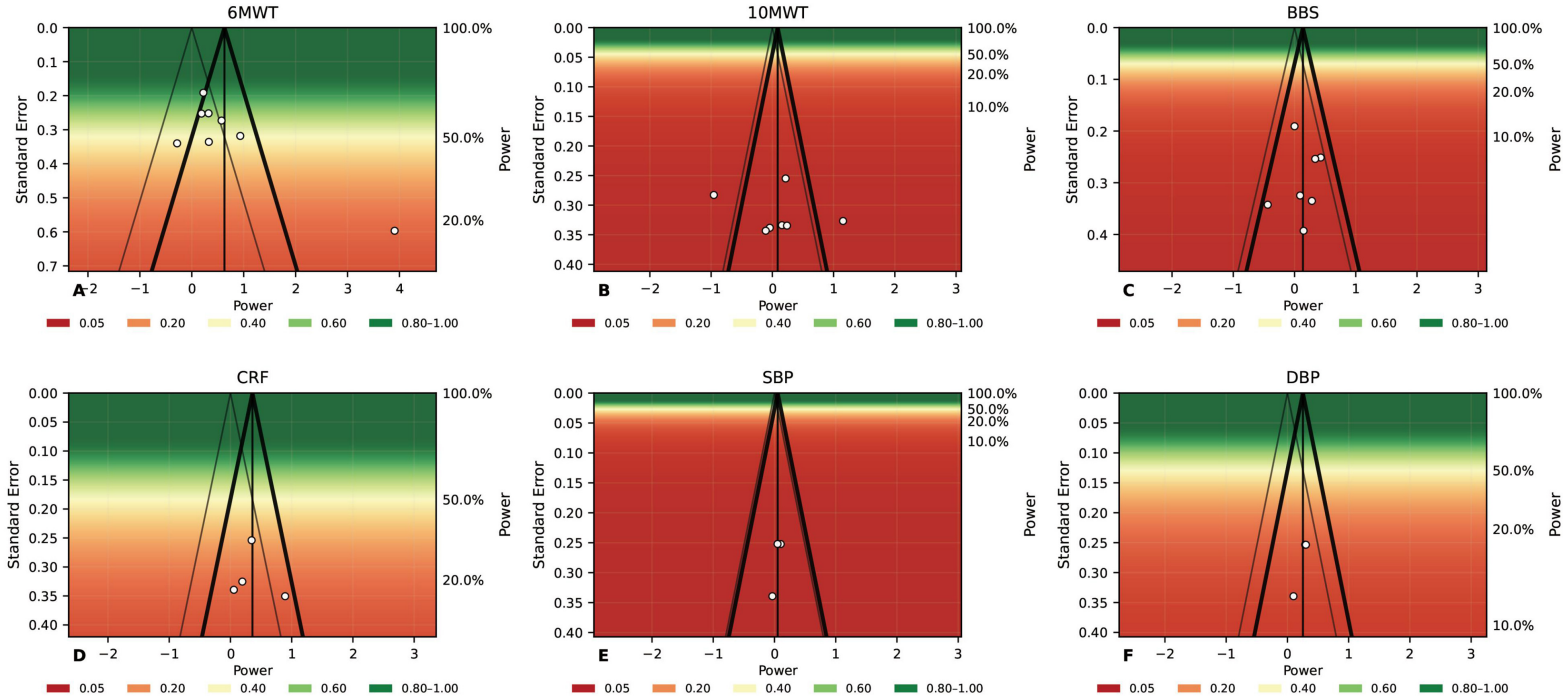
Sunset plots for publication-bias assessment: **(A)** 6-min walk test (6MWT); **(B)** 10-meter walk test (10MWT); **(C)** Berg Balance Scale (BBS); **(D)** cardiorespiratory fitness (CRF); **(E)** systolic blood pressure (SBP); and **(F)** diastolic blood pressure (DBP).

Publication bias and small-study effects were assessed using contour-enhanced funnel plots and Egger's regression tests when sufficient data were available. Because of the small number of studies contributing to the Scandinavian Stroke Scale outcome, publication bias could not be meaningfully assessed for this outcome (Table 6). For the remaining outcomes, Egger's regression tests did not detect clear evidence of small-study effects. The *p*-values for the bias coefficient were as follows: systolic blood pressure, *p* = 0.234; diastolic blood pressure, *p* = 0.193; cardiorespiratory fitness, *p* = 0.871; BBS, *p* = 0.753; 6MWT, *p* = 0.225; and 10MWT, *p* = 0.776. However, these findings should not be interpreted as evidence that publication bias was absent. Egger's test has low statistical power when the number of studies or effect sizes is small, and several outcomes in this review were based on sparse data (Figures 6, 7). Therefore, the appropriate interpretation is that no clear evidence of small-study effects was detected, but the assessment was underpowered.

**Table 6:** Meta-analysis of Egger's test results.

Outcome	Std_Eff	Coef.	Std. err	*t*	*p* > |*t*|	[95% conf. interval]
SBP	Slope	0.398	0.129	3.082	0.200	−1.242, 2.038
	Bias	−1.301	0.502	−2.591	0.234	−7.679, 5.077
DBP	Slope	0.771	0.157	4.909	0.128	−1.225, 2.767
	Bias	−1.946	0.608	−3.203	0.193	−9.668, 5.775
CRF	Slope	0.127	1.211	0.105	0.926	−5.082, 5.336
	bias	0.749	4.067	0.184	0.871	−16.749, 18.247
BBS	Slope	0.417	0.417	−0.010	0.992	−1.077, 1.068
	bias	1.705	1.705	0.333	0.753	−3.815, 4.949
6WMT	Slope	0.534	0.534	−0.735	0.490	−1.700, 0.915
	bias	2.176	2.176	1.352	0.225	−2.383, 8.267
10WMT	Slope	1.850	1.850	−0.275	0.794	−5.263, 4.246
	bias	6.225	6.225	0.300	0.776	−14.137, 17.868

### 3.8 GRADE evidence quality evaluation

The certainty of evidence was rated as low for BBS, SBP, and DBP, and very low for 6MWT, 10MWT, cardiorespiratory fitness, and SSS. The main reasons for downgrading were risk of bias, inconsistency, imprecision, and limited outcome-specific evidence. For BBS, the evidence was rated as low because the effect was small and the lower bound of the confidence interval was close to the null value (Table 7). For 6MWT, although the pooled average effect was statistically significant, the certainty of evidence was very low because of methodological concerns and heterogeneity. For 10MWT, the estimate was imprecise and statistically non-significant. For cardiorespiratory fitness and SSS, certainty was limited by sparse data and imprecision. For SBP and DBP, evidence certainty was low and pooled effects were not statistically significant. Taken together, the GRADE assessment indicates that current evidence regarding HIIT after stroke remains limited. Therefore, the clinical implications of the pooled effects should be interpreted cautiously, and stronger conclusions require adequately powered randomized trials with standardized HIIT definitions, rigorous adverse-event monitoring, and longer follow-up. Blood-pressure responses to HIIT may vary by population, baseline blood pressure, and comparator intensity, as shown in prior reviews of hypertensive and cardiovascular populations (64, 65).

**Table 7:** GRADE evidence quality assessment of the included studies.

Outcome	No. of studies	Quality assessment			Participants			*ES (*95%CI)	Certainty of evidence
		Risk of bias	Inconsistency	Indirectness	Imprecision	Intervention	Control		
SSS	2	Serious	Not serious	Not serious	Very serious	81	73	0.29 (−0.04 to 0.62)	⊕°°°
									Very low
SBP	3	Not serious	Not serious	Not serious	Very serious	81	80	0.05 (−0.25 to 0.36)	⊕⊕°°
									Low
DBP	3	Not serious	Not serious	Not serious	Very serious	81	80	0.25 (−0.05 to 0.56)	⊕⊕°°
									Low
CRF	4	Serious	Not serious	Not serious	Very serious	88	84	0.36 (0.05 to 0.66)	⊕°°°
									Very low
BBS	8	Serious	Not serious	Not serious	Serious	187	186	0.20 (0.01 to 0.39)	⊕⊕°°
									Low
6MWT	8	Serious	Very serious	Not serious	Not serious	222	220	0.41 (0.22 to 0.61)	⊕°°°
									Very low
10MWT	6	Serious	Very serious	Not serious	Serious	135	132	0.06 (−0.16 to 0.29)	⊕°°°
									Very low

## 4 Discussion

Balance dysfunction is one of the most common and clinically significant functional impairments in stroke survivors, closely related to fall risk, independent walking ability, and activities of daily living (12, 13). The Berg Balance Scale (BBS) assesses both static and dynamic postural control through multi-task situations such as sit-to-stand transitions, turning, reaching, and single-leg standing. It is simple to administer, highly reproducible, and sensitive to functional changes, making it a commonly used tool to evaluate balance rehabilitation outcomes post-stroke (14). The results from our three-level meta-analysis suggested a small favorable effect of HIIT on BBS (ES = 0.20, 95% CI 0.01 to 0.39, *p* = 0.039), indicating that HIIT may improve balance performance after stroke. However, given the small magnitude of effect and low certainty of evidence, this finding should be interpreted cautiously (15). Potential explanations may include improvements in cardiopulmonary capacity, lower-limb muscle performance, and neuromuscular control; however, these mechanisms were not directly tested in the present meta-analysis and therefore remain inferential rather than confirmatory (16, 17). Recent network meta-analytic evidence also suggests that different exercise interventions may have differential effects on balance and related functions after stroke (58).

Given the variations in intervention protocols and control groups across studies, HIIT is currently more suitable as a potential adjunct to conventional balance training rather than as a definitive replacement for established rehabilitation approaches. Individualized intensity control and safety monitoring remain important when applying HIIT in post-stroke rehabilitation (18). Subgroup analysis suggested that training frequencies ≤ 3 sessions/week, intervention durations ≥5 weeks, session durations < 30 min, and weekly total training volume < 600 MET min/week showed numerically different improvement trends. However, these findings were exploratory and based on a limited number of studies and multiple comparisons. Therefore, they should not be interpreted as evidence of an optimal HIIT prescription. In particular, the observation that shorter sessions were associated with larger numerical effects should be regarded as a preliminary signal that may be worth testing in future dose-finding trials, rather than evidence that a session duration of less than 30 min is optimal (19). Therefore, stroke rehabilitation using HIIT should be individualized, and future studies should further examine whether specific session durations or training volumes are associated with better balance outcomes under standardized protocols (20).

From a sensory and proprioceptive-neural control perspective, common post-stroke impairments such as sensory input integration deficits, reduced lower-limb proprioception, and delayed neuromuscular responses may contribute to unstable postural control strategies, impaired center-of-mass regulation, and balance instability during static and dynamic tasks (21). HIIT may provide a relatively strong physiological and neuromuscular stimulus, which could theoretically support motor unit recruitment and postural adjustment capacity. Nevertheless, because the included trials did not directly measure neural recruitment, proprioceptive recovery, or sensory-motor integration, these explanations should be considered plausible mechanisms rather than confirmed causal pathways (22, 23).

From a biomechanical and kinetic-chain perspective, post-stroke patients often experience lower-limb weakness, impaired muscle coordination, and gait compensation, leading to decreased stability during the support phase, a narrowed range of center-of-mass control, and increased fall risk. HIIT may improve lower-limb strength reserve, exercise tolerance, and trunk-lower limb coordination, which could indirectly contribute to improved performance on functional balance tasks (24). However, the present meta-analysis cannot determine whether the observed BBS improvement was caused by biomechanical adaptation, neural control changes, cardiopulmonary improvement, or other rehabilitation-related factors. In summary, HIIT may contribute to balance improvement through several plausible pathways, including cardiopulmonary and neuromuscular adaptation, sensory-motor integration, and kinetic-chain coordination, but these mechanisms require direct testing in future trials (25). Based on the subgroup results and the fatigue susceptibility of stroke patients, shorter-session HIIT protocols combined with conventional balance training may be worth further investigation, but they should not be interpreted as an established clinical prescription.

The 6-min walk test (6MWT) and 10-meter walk test (10MWT) focus on walking endurance and short-distance walking speed or gait-control quality, respectively. Together, they are important indicators of post-stroke mobility (26, 27). The 6MWT primarily reflects submaximal walking endurance and exercise capacity, providing a representation of activity tolerance and participation in community settings (28, 29). The 10MWT emphasizes walking speed output and gait stability over short distances, and walking speed is often considered a clinically relevant functional indicator closely related to activity levels and prognosis (30, 31).

Our three-level meta-analysis suggested that HIIT may improve 6MWT performance (ES = 0.41, 95% CI 0.22 to 0.61, *p* < 0.001), indicating a favorable average effect on walking endurance and sustained walking capacity in stroke survivors. Sensitivity analysis indicated that this average effect did not depend on any single study; however, this does not eliminate the presence of heterogeneity. Further regression analysis suggested that publication region was associated with 6MWT effect size (*p* = 0.042), but this finding should be interpreted cautiously because region may be confounded by rehabilitation setting, study design, intervention modality, comparator condition, baseline severity, achieved intensity, adherence, sample size, and publication year (32). Mechanistically, the improvement in 6MWT may be consistent with cardiopulmonary adaptation, peripheral oxygen utilization, walking economy, and fatigue resistance induced by high-intensity interval exercise (33, 34). These interpretations are consistent with literature on HIIT versus moderate-intensity continuous training and exercise-related biomarkers or adaptations, although direct evidence in post-stroke populations remains limited (57, 59, 61, 63). These findings are broadly consistent with previous literature suggesting that aerobic or high-intensity training may improve walking capacity and physical fitness in stroke patients, although uncertainty remains regarding optimal prescription and patient selection (13, 35, 36).

In contrast, the pooled effect for 10MWT was not statistically significant (ES = 0.06, 95% CI−0.16 to 0.29, *p* = 0.579). This suggests that current evidence does not demonstrate a clear effect of HIIT on short-distance walking speed or gait-control performance. This result may be related to the specific characteristics of the 10MWT: short-distance gait speed depends not only on physical capacity but also on gait coordination, lower-limb symmetry, trunk control, step timing, confidence, and fine-tuning of gait strategies (37). Furthermore, fatigue induced by high-intensity exercise may affect movement quality and speed output in some stroke survivors, thereby limiting transfer to short-distance speed-based tasks (38). Notably, some included studies suggested that low-intensity interval training might show numerically favorable effects on 10MWT compared with HIIT. However, this finding was based on limited studies and sample sizes and may be influenced by intensity definitions, adherence, baseline function, and the adequacy of gait-quality reporting. Therefore, this result should be considered hypothesis-generating, and further research with standardized intensity prescriptions and outcome measures is required (12, 38, 39). Overall, current evidence suggests that HIIT may be more relevant for endurance-based walking capacity than for short-distance gait speed or gait-control performance. Clinically, HIIT may be considered as a promising adjunctive strategy for enhancing exercise capacity and endurance, but improvements in gait speed and gait quality may require additional task-specific gait training, symmetry training, balance training, and movement-quality feedback (40, 41).

Risk of bias and certainty of evidence should be central to the interpretation of these findings. Only two of the 14 included trials were judged as low risk of bias overall, whereas nine had some concerns and three were judged as high risk. In addition, GRADE certainty ranged from low to very low across outcomes. These limitations reduce confidence in the estimated effects and indicate that the current evidence should be viewed as suggestive rather than definitive. Therefore, HIIT should be regarded as a promising adjunctive rehabilitation strategy rather than a firmly established rehabilitation prescription. Future adequately powered randomized trials should use standardized HIIT definitions, report achieved intensity and adherence, monitor adverse events systematically, include longer follow-up, and directly compare HIIT with moderate-intensity continuous training, low-intensity interval training, and task-specific balance or gait interventions.

## 5 Limitations

(1) Some studies included in this review did not report details on allocation concealment or blinding procedures, which may decrease the reliability of the results or introduce subjective bias.

(2) There were significant variations in the HIIT intervention protocols across studies in terms of intensity definition, interval structure, training frequency, duration, and total training volume. Additionally, the control group interventions varied, which may increase heterogeneity between studies and affect the stability of the pooled effect.

(3) Differences in the outcome measures and measurement protocols across studies were observed, with some outcomes having fewer studies included. Additionally, the stability of the 10MWT outcome was relatively insufficient, which may lead to bias in effect size estimation and a decrease in the certainty of the evidence.

## 6 Conclusions

(1) Low- to very-low-certainty evidence suggests that high-intensity interval training (HIIT) may improve balance ability, walking endurance, and cardiorespiratory fitness in post-stroke patients. However, the effects of HIIT on short-distance walking speed or gait-control performance, blood pressure, and stroke severity remain uncertain.

(2) The subgroup and meta-regression findings should not be interpreted as evidence of optimal rehabilitation protocols. The observed patterns related to training frequency, intervention duration, session duration, and weekly training volume should be regarded only as exploratory dose-parameter signals that require confirmation in future studies.

(3) Clinically, HIIT may be considered a promising adjunctive strategy to conventional post-stroke rehabilitation, provided that individualized intensity control, safety screening, and adverse-event monitoring are implemented. Current evidence is not sufficient to support HIIT as a definitive or standardized rehabilitation prescription.

(4) Future adequately powered randomized controlled trials should use standardized HIIT definitions, report adherence and achieved training intensity, systematically monitor adverse events, include longer follow-up, stratify participants by stroke stage and baseline severity, and directly compare HIIT with moderate-intensity continuous training, low-intensity interval training, and task-specific gait or balance training.

## Data Availability

The raw data supporting the conclusions of this article will be made available by the authors, without undue reservation.

## References

[1] Chutinet A Charnnarong C Suwanwela NC. Stroke from infection. Cerebrovasc Dis Extra. (2025) 15:118–29. doi: 10.1159/000544986 40068656 PMC12237291

[2] Khan F Chevidikunnan MF. Prevalence of balance impairment and factors associated with balance among patients with stroke: a cross sectional retrospective case control study. Healthcare. (2021) 9:320. doi: 10.3390/healthcare9030320 33805643 PMC7998930

[3] Xu F Soh KG Chan YM Bai XR Qi F Deng N. Effects of tai chi on postural balance and quality of life among the elderly with gait disorders: a systematic review. PLoS ONE. (2023) 18:e0287035. doi: 10.1371/journal.pone.0287035 37768953 PMC10538728

[4] Mehrholz J Kugler J Pohl M Elsner B. Electromechanical-assisted training for walking after stroke. Cochrane Database Syst Rev. (2025) 5:CD006185. Published 2025 May 14. doi: 10.1002/14651858.CD006185.pub6 40365867 PMC12076539

[5] Forster A Young J. Incidence and consequences of falls due to stroke: a systematic inquiry. BMJ. (1995) 311:83–6. doi: 10.1136/bmj.311.6997.83 7613406 PMC2550147

[6] Krohn M Rintala A Immonen J Sjögren T. The effectiveness of therapeutic exercise interventions with virtual reality on balance and walking among persons with chronic stroke: systematic review, meta-analysis, and meta-regression of randomized controlled trials. J Med Internet Res. (2024) 26:e59136. doi: 10.2196/59136 39621381 PMC11650088

[7] Saunders DH Sanderson M Hayes S Johnson L Kramer S Carter DD . Physical fitness training for stroke patients. Cochrane Database Syst Rev. (2020) 3:CD003316. doi: 10.1002/14651858.CD003316.pub7 32196635 PMC7083515

[8] Blatgé H Paul L van Wijck F. High-intensity interval training after stroke: a mixed-methods systematic review and meta-analysis of safety, feasibility and acceptability. Clin Rehabil. (2025) 40:304–37. doi: 10.1177/02692155251385222 41232083 PMC12920696

[9] Gjellesvik TI Becker F Tjønna AE Indredavik B Lundgaard E Solbakken H . Effects of high-intensity interval training after stroke (the hiit stroke study) on physical and cognitive function: a multicenter randomized controlled trial. Arch Phys Med Rehabil. (2021) 102:1683–91. doi: 10.1016/j.apmr.2021.05.008 34102144

[10] Meulenkamp B Stacey D Fergusson D Hutton B Mlis RS Graham ID. Protocol for treatment of Achilles tendon ruptures; a systematic review with network meta-analysis. Syst Rev. (2018) 7:247. doi: 10.1186/s13643-018-0912-5 30580763 PMC6304227

[11] Page MJ McKenzie JE Bossuyt PM Boutron I Hoffmann TC Mulrow CD . The PRISMA 2020 statement: an updated guideline for reporting systematic reviews. BMJ. (2021) 372:n71. doi: 10.1136/bmj.n71 33782057 PMC8005924

[12] Vahlberg B Cederholm T Lindmark B Zetterberg L Hellström K. Short-term and long-term effects of a progressive resistance and balance exercise program in individuals with chronic stroke: a randomized controlled trial. Disabil Rehabil. (2017) 39:1615–22. doi: 10.1080/09638288.2016.1206631 27415645

[13] Luo Y Chen X Gong H Chen L Zhang L Li S. Efficacy of aerobic exercises for knee osteoarthritis: a network meta analysis of randomized clinical trials. J Orthop Surg Res. (2025) 20:557. doi: 10.1186/s13018-025-05973-z 40452037 PMC12128381

[14] Berg KO Wood-Dauphinee SL Williams JI Maki B. Measuring balance in the elderly: validation of an instrument. Can J Public Health. (1992) 83:S7–11. 1468055

[15] Blum L Korner-Bitensky N. Usefulness of the berg balance scale in stroke rehabilitation: a systematic review. Phys Ther. (2008) 88:559–66. doi: 10.2522/ptj.20070205 18292215

[16] Wang CY Hsieh CL Olson SL Wang CH Sheu CF Liang CC. Psychometric properties of the Berg Balance Scale in a neurorehabilitation population. J Rehabil Med. (2006) 38:238–44. 10.1016/S0929-6646(09)60283-7 17185241

[17] Anjos JM Neto MG Dos Santos FS Almeida KO Bocchi EA Lima Bitar YS . The impact of high-intensity interval training on functioning and health-related quality of life in post-stroke patients: a systematic review with meta-analysis. Clin Rehabil. (2022) 36:726–39. doi: 10.1177/02692155221087082 35290104

[18] Billinger SA Arena R Bernhardt J Eng JJ Franklin BA Johnson CM . Physical activity and exercise recommendations for stroke survivors: a scientific statement from the American heart association/american stroke association. Stroke. (2014) 45:2532–53. doi: 10.1161/STR.0000000000000022 24846875

[19] Mansfield A Inness EL Danells CJ Jagroop D Bhatt T Huntley AH. Determining the optimal dose of reactive balance training after stroke: study protocol for a pilot randomised controlled trial. BMJ Open. (2020) 10:e038073. doi: 10.1136/bmjopen-2020-038073 32847916 PMC7451480

[20] Horak FB. Postural orientation and equilibrium: what do we need to know about neural control of balance to prevent falls? Age and Ageing. (2006) 35:ii7–ii11. doi: 10.1093/ageing/afl077 16926210

[21] Cho JE Kim H. Ankle proprioception deficit is the strongest factor predicting balance impairment in patients with chronic stroke. Arch Rehabil Res Clin Transl. (2021) 3:100165. doi: 10.1016/j.arrct.2021.100165 34977547 PMC8683870

[22] Yu Y Yan X Chen C . Correlation between proprioceptive impairment and motor deficits after stroke: a cross-sectional study. Front Neurol. (2022) 13:688616. doi: 10.3389/fneur.2021.688616 35095706 PMC8793362

[23] Montero-Almagro G Bernal-Utrera C Geribaldi-Doldán N Nunez-Abades P Castro C Rodriguez-Blanco C. Influence of high-intensity interval training on neuroplasticity markers in post-stroke patients: systematic review. J Clin Med. (2024) 13:1985. doi: 10.3390/jcm13071985 38610750 PMC11012260

[24] Ashcroft SK Ironside DD Johnson L Kuys SS Thompson-Butel AG. Effect of exercise on brain-derived neurotrophic factor in stroke survivors: a systematic review and meta-analysis. Stroke. (2022) 53:3706–16. doi: 10.1161/STROKEAHA.122.039919 36278401

[25] Liu H Yin H Yi Y Liu C Li C. Effects of different rehabilitation training on balance function in stroke patients: a systematic review and network meta-analysis. Arch Med Sci. (2023) 19:1671–83. doi: 10.5114/aoms/167385 38058731 PMC10696991

[26] Busk H Holm P Skou ST Seitner S Siemsen T Wienecke T. Inter-rater reliability and agreement of 6 minute walk test and 10 meter walk test at comfortable walk speed in patients with acute stroke. Physiother Theory Pract. (2023) 39:1024–32. doi: 10.1080/09593985.2022.2030830 35109744

[27] American Thoracic Society. ATS statement: guidelines for the six-minute walk test. Am J Respir Crit Care Med. (2002) 166:111–7. doi: 10.1164/ajrccm.166.1.at1102 12091180

[28] Fulk G D He Y. Minimal clinically important difference of the 6-minute walk test in people with stroke. J Neurol Phys Ther. (2018) 42:235–40. doi: 10.1097/NPT.0000000000000236 30138230

[29] Tang A Eng JJ Rand D. Relationship between perceived and measured changes in walking after stroke. J Neurol Phys Ther. (2012) 36:115–21. doi: 10.1097/NPT.0b013e318262dbd0 22850336 PMC3501529

[30] Tilson JK Sullivan KJ Cen SY Rose DK Koradia CH Azen SP . Meaningful gait speed improvement during the first 60 days poststroke: minimal clinically important difference. Phys Ther. (2010) 90:196–208. doi: 10.2522/ptj.20090079 20022995 PMC2816032

[31] Fritz S Lusardi M. White paper: “walking speed: the sixth vital sign”. J Geriatr Phys Ther. (2009) 32:46–9. doi: 10.1519/00139143-200932020-00002 20039582

[32] Moncion K Rodrigues L De Las Heras B Noguchi KS Wiley E Eng JJ . Cardiorespiratory fitness benefits of high-intensity interval training after stroke: a randomized controlled trial. Stroke. (2024) 55:2202–11. doi: 10.1161/STROKEAHA.124.046564 39113181

[33] Weston K S Wisløff U Coombes J S. High-intensity interval training in patients with lifestyle-induced cardiometabolic disease: a systematic review and meta-analysis. British J Sports Med. (2014) 48:1227–34. doi: 10.1136/bjsports-2013-092576 24144531

[34] Coletti C Acosta GF Keslacy S Coletti D. Exercise-mediated reinnervation of skeletal muscle in elderly people: an update. Eur J Transl Myol. (2022) 32:10416. doi: 10.4081/ejtm.2022.10416 35234025 PMC8992679

[35] Wiener J McIntyre A Janssen S Chow JT Batey C Teasell R. Effectiveness of high-intensity interval training for fitness and mobility post stroke: a systematic review. PM R. (2019) 11:868–78. doi: 10.1002/pmrj.12154 30859720

[36] Barber M Fail M Shields M Stott DJ Langhorne P. Validity and reliability of estimating the scandinavian stroke scale score from medical records. Cerebrovasc Dis. (2004) 17:224–7. doi: 10.1159/000075795 14707426

[37] Goh HT Stewart JC. Poststroke fatigue is related to motor and cognitive performance: a secondary analysis. J Neurol Phys Ther. (2019) 43:233–9. doi: 10.1097/NPT.0000000000000290 31436613 PMC8130858

[38] Usman JS Wong TWL Ng SSM. Relationships of post-stroke fatigue with mobility, recovery, performance, and participation-related outcomes: a systematic review and meta-analysis. Front Neurol. (2024)15:1420443. doi: 10.3389/fneur.2024.1420443 PMC11493601 39440256

[39] Baricich A Borg MB Battaglia M Facciorusso S Spina S Invernizzi M . High-intensity exercise training impact on cardiorespiratory fitness, gait ability, and balance in stroke survivors: a systematic review and meta-analysis. J Clin Med. (2024) 13:5498. doi: 10.3390/jcm13185498 39336984 PMC11432212

[40] Lee M-H Lee D-Y. Effects of task-oriented training on gait outcomes and balance in individuals with stroke: systematic review and meta-analysis of RCTs. J Clin Med. (2025) 14:8766. doi: 10.3390/jcm14248766 41464666 PMC12734183

[41] Hill G Johnson F Uy J Serrada I Benyamin B Van Den Berg M . Moderate intensity aerobic exercise may enhance neuroplasticity of the contralesional hemisphere after stroke: a randomised controlled study. Sci Rep. (2023) 13:14440. doi: 10.1038/s41598-023-40902-2 37660093 PMC10475034

[42] Amanzonwé ER Noukpo SI Adoukonou T Bonnechére B Feys P Hansen D . Exercise intensity matters in the rehabilitation of stroke in the acute stage: a randomized controlled trial. Neurorehabil Neural Repair. (2025) 39:892–905. doi: 10.1177/15459683251356969 40788139

[43] Gjellesvik TI Becker F Tjønna AE Indredavik B Nilsen H Brurok B . Effects of high-intensity interval training after stroke (the hiit-stroke study): a multicenter randomized controlled trial. Arch Phys Med Rehabil. (2020) 101:939–47. doi: 10.1016/j.apmr.2020.02.006 32145280

[44] Globas C Becker C Cerny J Lam JM Lindemann U Forrester LW . Chronic stroke survivors benefit from high-intensity aerobic treadmill exercise: a randomized control trial. Neurorehabil Neural Repair. (2012) 26:85–95. doi: 10.1177/1545968311418675 21885867

[45] Hornby TG Rafferty MR Pinto D French D Jordan N. Cost-effectiveness of high-intensity training vs conventional therapy for individuals with subacute stroke. Arch Phys Med Rehabil. (2022) 103:S197–204. doi: 10.1016/j.apmr.2021.05.017 34228956 PMC12761790

[46] Krawcyk RS Vinther A Petersen NC Faber J Iversen HK Christensen T . High-intensity training in patients with lacunar stroke: a one-year follow-up. J Stroke Cerebrovasc Dis. (2023) 32:106973. doi: 10.1016/j.jstrokecerebrovasdis.2022.106973 36623990

[47] Lamberti N Straudi S Malagoni AM Argiró M Felisatti M Nardini E . Effects of low-intensity endurance and resistance training on mobility in chronic stroke survivors: a pilot randomized controlled study. Eur J Phys Rehabil Med. (2017) 53:228–39. doi: 10.23736/S1973-9087.16.04322-7 27626795

[48] Lapointe T Houle J Sia YT Payette M Trudeau F. Addition of high-intensity interval training to a moderate intensity continuous training cardiovascular rehabilitation program after ischemic cerebrovascular disease: a randomized controlled trial. Front Neurol. (2023) 13:963950. doi: 10.3389/fneur.2022.963950 36686521 PMC9846748

[49] Lau KW Mak MK. Speed-dependent treadmill training is effective to improve gait and balance performance in patients with sub-acute stroke. J Rehabil Med. (2011) 43:709–13. doi: 10.2340/16501977-0838 21698340

[50] Moore JL Nordvik JE Erichsen A Rosseland I Bø E Hornby TG . Implementation of high-intensity stepping training during inpatient stroke rehabilitation improves functional outcomes. Stroke. (2020) 51:563–70. doi: 10.1161/STROKEAHA.119.027450 31884902 PMC7034641

[51] Pang MY Eng JJ Dawson AS McKay HA Harris JE. A community-based fitness and mobility exercise program for older adults with chronic stroke: a randomized, controlled trial. J Am Geriatr Soc. (2005) 53:1667–74. doi: 10.1111/j.1532-5415.2005.53521.x 16181164 PMC3226792

[52] Ivey FM Stookey AD Hafer-Macko CE Ryan AS Macko RF. Higher treadmill training intensity to address functional aerobic impairment after stroke. J Stroke Cerebrovasc Dis. (2015) 24:2539–46. doi: 10.1016/j.jstrokecerebrovasdis.2015.07.002 26303787 PMC4908456

[53] Sandberg K Kleist M Falk L Enthoven P. Effects of twice-weekly intense aerobic exercise in early subacute stroke: a randomized controlled trial. Arch Phys Med Rehabil. (2016) 97:1244–53. doi: 10.1016/j.apmr.2016.01.030 26903147

[54] Steen Krawcyk R Vinther A Petersen NC Faber J Iversen HK Christensen T . Effect of home-based high-intensity interval training in patients with lacunar stroke: a randomized controlled trial. Front Neurol. (2019) 10:664. doi: 10.3389/fneur.2019.00664 31316451 PMC6611174

[55] Zaman T Batool U Hassan RM Bashir F Aroofa H Noor F . Effects of high-intensity interval training on muscle strength, atrophy and aerobic capacity in stroke patients. J Popul Ther Clini Pharmacol. (2025). doi: 10.53555/70x65h09

[56] Askim T Dahl AE Aamot IL Hokstad A Helbostad J Indredavik B. High-intensity aerobic interval training for patients 3-9 months after stroke: a feasibility study. Physiother Res Int. (2014) 19:129–39. doi: 10.1002/pri.1573 24375978

[57] Guo Z Li M Cai J Gong W Liu Y Liu Z. Effect of high-intensity interval training vs. moderate-intensity continuous training on fat loss and cardiorespiratory fitness in the young and middle-aged a systematic review and meta-analysis. Int J Environ Res Public Health. (2023) 20:4741. doi: 10.3390/ijerph20064741 36981649 PMC10048683

[58] Du M Chen L Xia L Li Y Ma E Hu Z . Effectiveness of different exercise interventions on balance and cognitive functions in stroke patients: a network meta-analysis. BMC Sports Sci Med Rehabil. (2025) 17:250. doi: 10.1186/s13102-025-01267-3 40866917 PMC12382136

[59] Limaye NS Carvalho LB Kramer S. Effects of aerobic exercise on serum biomarkers of neuroplasticity and brain repair in stroke: a systematic review. Arch Phys Med Rehabil. (2021) 102:1633–44. doi: 10.1016/j.apmr.2021.04.010 33992633

[60] Drevon D Fursa SR Malcolm AL. Intercoder reliability and validity of WebPlotDigitizer in extracting graphed data. Behav Modif. (2017) 41:323–39. doi: 10.1177/0145445516673998 27760807

[61] Mueller S Winzer EB Duvinage A Gevaert AB Edelmann F Haller B . Effect of high-intensity interval training, moderate continuous training, or guideline-based physical activity advice on peak oxygen consumption in patients with heart failure with preserved ejection fraction: a randomized clinical trial. JAMA. (2021) 325:542–51. doi: 10.1001/jama.2020.26812 33560320 PMC7873782

[62] Wu X Yang D Zhu Q Xiao Y Huang H. The safety and efficacy of high-intensity interval training (HIIT) in post-stroke patients with moderate functional impairment: a systematic review and meta-analysis. Front Neurol. (2025) 16:1695243. doi: 10.3389/fneur.2025.1695243 41356240 PMC12675936

[63] MacInnis MJ Gibala MJ. Physiological adaptations to interval training and the role of exercise intensity. J Physiol. (2017) 595:2915–30. doi: 10.1113/JP273196 27748956 PMC5407969

[64] Li L Liu X Shen F Xu N Li Y Xu K . Effects of high-intensity interval training versus moderate-intensity continuous training on blood pressure in patients with hypertension: a meta-analysis. Medicine (Baltimore). (2022) 101:e32246. doi: 10.1097/MD.0000000000032246 36550888 PMC9771301

[65] Costa EC Hay JL Kehler DS Boreskie KF Arora RC Umpierre D . Effects of high-intensity interval training versus moderate-intensity continuous training on blood pressure in adults with pre- to established hypertension: a systematic review and meta-analysis of randomized trials. Sports Med. (2018) 48:2127–42. doi: 10.1007/s40279-018-0944-y 29949110

