# Supplementary material for: High-intensity interval training after stroke: a three-level random-effects meta-analysis with cluster-robust inference and exploratory dose-parameter signals
Source: Front Neurol. 2026 Jul 3;17:1804120. doi: 10.3389/fneur.2026.1804120 (PMC13375884; doi:10.3389/fneur.2026.1804120)
Supplement: Supplementary file 1 [file Table_1.docx]

**S1 Table. Detailed search strategy**

**Supplementary table S1.1. Search terms for query**

| Set | Search terms for query |
| --- | --- |
| #1 | stroke OR "cerebrovascular accident" OR CVA OR poststroke OR "post-stroke" OR "ischemic stroke" OR "ischaemic stroke" OR "hemorrhagic stroke" OR "haemorrhagic stroke" OR "brain ischemia" OR "intracranial hemorrhage" |
| #2 | "high-intensity interval training" OR "high intensity interval training" OR HIIT OR "high-intensity training" OR "high intensity training" OR "interval training" |
| #3 | "randomized controlled trial" OR random* OR trial OR controlled OR "clinical trial" OR placebo |
| #4 | "Berg Balance Scale" OR BBS OR balance OR "postural balance" |
| #5 | "6-minute walk" OR "six-minute walk" OR 6MWT OR "walk test" |
| #6 | "10-meter walk" OR "10 m walk" OR 10MWT OR "gait speed" OR "walking speed" |
| #7 | "blood pressure" OR hypertension OR SBP OR DBP OR "systolic blood pressure" OR "diastolic blood pressure" |
| #8 | "cardiorespiratory fitness" OR VO2peak OR "VO2 peak" OR VO2max OR "VO2 max" OR "peak oxygen uptake" |
| #9 | "Scandinavian Stroke Scale" OR SSS OR NIHSS OR "stroke severity" |
| #10 | mobility OR gait OR walking OR locomotion OR "functional capacity" |
| E1 | #1 AND #2 AND #3 AND #4 |
| E2 | #1 AND #2 AND #3 AND #5 |
| E3 | #1 AND #2 AND #3 AND #6 |
| E4 | #1 AND #2 AND #3 AND #7 |
| E5 | #1 AND #2 AND #3 AND #8 |
| E6 | #1 AND #2 AND #3 AND #9 |
| E7 | #1 AND #2 AND #3 AND #10 |

**Supplementary table S1.2. Search terms for query**

| Database | Strategy | Search strategy details |
| --- | --- | --- |
| PubMed | E1 | (stroke OR "cerebrovascular accident" OR CVA OR poststroke OR "post-stroke" OR "ischemic stroke" OR "hemorrhagic stroke") AND ("high-intensity interval training" OR HIIT OR "interval training" OR "high-intensity training") AND ("randomized controlled trial" OR random* OR trial OR controlled) AND ("Berg Balance Scale" OR BBS OR balance) AND English AND Humans |
| PubMed | E2 | (stroke OR "cerebrovascular accident" OR CVA OR poststroke OR "post-stroke" OR "ischemic stroke" OR "hemorrhagic stroke") AND ("high-intensity interval training" OR HIIT OR "interval training" OR "high-intensity training") AND ("randomized controlled trial" OR random* OR trial OR controlled) AND ("6-minute walk" OR 6MWT) AND English AND Humans |
| PubMed | E3 | (stroke OR "cerebrovascular accident" OR CVA OR poststroke OR "post-stroke" OR "ischemic stroke" OR "hemorrhagic stroke") AND ("high-intensity interval training" OR HIIT OR "interval training" OR "high-intensity training") AND ("randomized controlled trial" OR random* OR trial OR controlled) AND ("10-meter walk" OR 10MWT OR "gait speed") AND English AND Humans |
| PubMed | E4 | (stroke OR "cerebrovascular accident" OR CVA OR poststroke OR "post-stroke" OR "ischemic stroke" OR "hemorrhagic stroke") AND ("high-intensity interval training" OR HIIT OR "interval training" OR "high-intensity training") AND ("randomized controlled trial" OR random* OR trial OR controlled) AND ("blood pressure" OR hypertension OR SBP OR DBP) AND English AND Humans |
| PubMed | E5 | (stroke OR "cerebrovascular accident" OR CVA OR poststroke OR "post-stroke" OR "ischemic stroke" OR "hemorrhagic stroke") AND ("high-intensity interval training" OR HIIT OR "interval training" OR "high-intensity training") AND ("randomized controlled trial" OR random* OR trial OR controlled) AND ("cardiorespiratory fitness" OR VO2peak OR VO2max OR "peak oxygen uptake") AND English AND Humans |
| PubMed | E6 | (stroke OR "cerebrovascular accident" OR CVA OR poststroke OR "post-stroke" OR "ischemic stroke" OR "hemorrhagic stroke") AND ("high-intensity interval training" OR HIIT OR "interval training" OR "high-intensity training") AND ("randomized controlled trial" OR random* OR trial OR controlled) AND ("Scandinavian Stroke Scale" OR SSS OR NIHSS OR "stroke severity") AND English AND Humans |
| PubMed | E7 | (stroke OR "cerebrovascular accident" OR CVA OR poststroke OR "post-stroke" OR "ischemic stroke" OR "hemorrhagic stroke") AND ("high-intensity interval training" OR HIIT OR "interval training" OR "high-intensity training") AND ("randomized controlled trial" OR random* OR trial OR controlled) AND (mobility OR gait OR walking OR locomotion) AND English AND Humans |
| Web of Science | E1 | TS=((stroke OR "cerebrovascular accident" OR CVA OR poststroke OR "post-stroke" OR "ischemic stroke" OR "hemorrhagic stroke") AND ("high-intensity interval training" OR HIIT OR "interval training" OR "high-intensity training") AND ("randomized controlled trial" OR random* OR trial OR controlled) AND ("Berg Balance Scale" OR BBS OR balance)) AND LA=(English) |
| Web of Science | E2 | TS=((stroke OR "cerebrovascular accident" OR CVA OR poststroke OR "post-stroke" OR "ischemic stroke" OR "hemorrhagic stroke") AND ("high-intensity interval training" OR HIIT OR "interval training" OR "high-intensity training") AND ("randomized controlled trial" OR random* OR trial OR controlled) AND ("6-minute walk" OR 6MWT)) AND LA=(English) |
| Web of Science | E3 | TS=((stroke OR "cerebrovascular accident" OR CVA OR poststroke OR "post-stroke" OR "ischemic stroke" OR "hemorrhagic stroke") AND ("high-intensity interval training" OR HIIT OR "interval training" OR "high-intensity training") AND ("randomized controlled trial" OR random* OR trial OR controlled) AND ("10-meter walk" OR 10MWT OR "gait speed")) AND LA=(English) |
| Web of Science | E4 | TS=((stroke OR "cerebrovascular accident" OR CVA OR poststroke OR "post-stroke" OR "ischemic stroke" OR "hemorrhagic stroke") AND ("high-intensity interval training" OR HIIT OR "interval training" OR "high-intensity training") AND ("randomized controlled trial" OR random* OR trial OR controlled) AND ("blood pressure" OR hypertension OR SBP OR DBP)) AND LA=(English) |
| Web of Science | E5 | TS=((stroke OR "cerebrovascular accident" OR CVA OR poststroke OR "post-stroke" OR "ischemic stroke" OR "hemorrhagic stroke") AND ("high-intensity interval training" OR HIIT OR "interval training" OR "high-intensity training") AND ("randomized controlled trial" OR random* OR trial OR controlled) AND ("cardiorespiratory fitness" OR VO2peak OR VO2max OR "peak oxygen uptake")) AND LA=(English) |
| Web of Science | E6 | TS=((stroke OR "cerebrovascular accident" OR CVA OR poststroke OR "post-stroke" OR "ischemic stroke" OR "hemorrhagic stroke") AND ("high-intensity interval training" OR HIIT OR "interval training" OR "high-intensity training") AND ("randomized controlled trial" OR random* OR trial OR controlled) AND ("Scandinavian Stroke Scale" OR SSS OR NIHSS OR "stroke severity")) AND LA=(English) |
| Web of Science | E7 | TS=((stroke OR "cerebrovascular accident" OR CVA OR poststroke OR "post-stroke" OR "ischemic stroke" OR "hemorrhagic stroke") AND ("high-intensity interval training" OR HIIT OR "interval training" OR "high-intensity training") AND ("randomized controlled trial" OR random* OR trial OR controlled) AND (mobility OR gait OR walking OR locomotion)) AND LA=(English) |
| Embase | E1 | ((stroke OR "cerebrovascular accident" OR CVA OR poststroke OR "post-stroke" OR "ischemic stroke" OR "hemorrhagic stroke") AND ("high-intensity interval training" OR HIIT OR "interval training" OR "high-intensity training") AND ("randomized controlled trial" OR random* OR trial OR controlled) AND ("Berg Balance Scale" OR BBS OR balance)) :ti,ab,kw AND [english]/lim AND [humans]/lim |
| Embase | E2 | ((stroke OR "cerebrovascular accident" OR CVA OR poststroke OR "post-stroke" OR "ischemic stroke" OR "hemorrhagic stroke") AND ("high-intensity interval training" OR HIIT OR "interval training" OR "high-intensity training") AND ("randomized controlled trial" OR random* OR trial OR controlled) AND ("6-minute walk" OR 6MWT)) :ti,ab,kw AND [english]/lim AND [humans]/lim |
| Embase | E3 | ((stroke OR "cerebrovascular accident" OR CVA OR poststroke OR "post-stroke" OR "ischemic stroke" OR "hemorrhagic stroke") AND ("high-intensity interval training" OR HIIT OR "interval training" OR "high-intensity training") AND ("randomized controlled trial" OR random* OR trial OR controlled) AND ("10-meter walk" OR 10MWT OR "gait speed")) :ti,ab,kw AND [english]/lim AND [humans]/lim |
| Embase | E4 | ((stroke OR "cerebrovascular accident" OR CVA OR poststroke OR "post-stroke" OR "ischemic stroke" OR "hemorrhagic stroke") AND ("high-intensity interval training" OR HIIT OR "interval training" OR "high-intensity training") AND ("randomized controlled trial" OR random* OR trial OR controlled) AND ("blood pressure" OR hypertension OR SBP OR DBP)) :ti,ab,kw AND [english]/lim AND [humans]/lim |
| Embase | E5 | ((stroke OR "cerebrovascular accident" OR CVA OR poststroke OR "post-stroke" OR "ischemic stroke" OR "hemorrhagic stroke") AND ("high-intensity interval training" OR HIIT OR "interval training" OR "high-intensity training") AND ("randomized controlled trial" OR random* OR trial OR controlled) AND ("cardiorespiratory fitness" OR VO2peak OR VO2max OR "peak oxygen uptake")) :ti,ab,kw AND [english]/lim AND [humans]/lim |
| Embase | E6 | ((stroke OR "cerebrovascular accident" OR CVA OR poststroke OR "post-stroke" OR "ischemic stroke" OR "hemorrhagic stroke") AND ("high-intensity interval training" OR HIIT OR "interval training" OR "high-intensity training") AND ("randomized controlled trial" OR random* OR trial OR controlled) AND ("Scandinavian Stroke Scale" OR SSS OR NIHSS OR "stroke severity")) :ti,ab,kw AND [english]/lim AND [humans]/lim |
| Embase | E7 | ((stroke OR "cerebrovascular accident" OR CVA OR poststroke OR "post-stroke" OR "ischemic stroke" OR "hemorrhagic stroke") AND ("high-intensity interval training" OR HIIT OR "interval training" OR "high-intensity training") AND ("randomized controlled trial" OR random* OR trial OR controlled) AND (mobility OR gait OR walking OR locomotion)) :ti,ab,kw AND [english]/lim AND [humans]/lim |
| Scopus | E1 | TITLE-ABS-KEY((stroke OR "cerebrovascular accident" OR CVA OR poststroke OR "post-stroke" OR "ischemic stroke" OR "hemorrhagic stroke") AND ("high-intensity interval training" OR HIIT OR "interval training" OR "high-intensity training") AND ("randomized controlled trial" OR random* OR trial OR controlled) AND ("Berg Balance Scale" OR BBS OR balance)) AND (LIMIT-TO(LANGUAGE, "English")) |
| Scopus | E2 | TITLE-ABS-KEY((stroke OR "cerebrovascular accident" OR CVA OR poststroke OR "post-stroke" OR "ischemic stroke" OR "hemorrhagic stroke") AND ("high-intensity interval training" OR HIIT OR "interval training" OR "high-intensity training") AND ("randomized controlled trial" OR random* OR trial OR controlled) AND ("6-minute walk" OR 6MWT)) AND (LIMIT-TO(LANGUAGE, "English")) |
| Scopus | E3 | TITLE-ABS-KEY((stroke OR "cerebrovascular accident" OR CVA OR poststroke OR "post-stroke" OR "ischemic stroke" OR "hemorrhagic stroke") AND ("high-intensity interval training" OR HIIT OR "interval training" OR "high-intensity training") AND ("randomized controlled trial" OR random* OR trial OR controlled) AND ("10-meter walk" OR 10MWT OR "gait speed")) AND (LIMIT-TO(LANGUAGE, "English")) |
| Scopus | E4 | TITLE-ABS-KEY((stroke OR "cerebrovascular accident" OR CVA OR poststroke OR "post-stroke" OR "ischemic stroke" OR "hemorrhagic stroke") AND ("high-intensity interval training" OR HIIT OR "interval training" OR "high-intensity training") AND ("randomized controlled trial" OR random* OR trial OR controlled) AND ("blood pressure" OR hypertension OR SBP OR DBP)) AND (LIMIT-TO(LANGUAGE, "English")) |
| Scopus | E5 | TITLE-ABS-KEY((stroke OR "cerebrovascular accident" OR CVA OR poststroke OR "post-stroke" OR "ischemic stroke" OR "hemorrhagic stroke") AND ("high-intensity interval training" OR HIIT OR "interval training" OR "high-intensity training") AND ("randomized controlled trial" OR random* OR trial OR controlled) AND ("cardiorespiratory fitness" OR VO2peak OR VO2max OR "peak oxygen uptake")) AND (LIMIT-TO(LANGUAGE, "English")) |
| Scopus | E6 | TITLE-ABS-KEY((stroke OR "cerebrovascular accident" OR CVA OR poststroke OR "post-stroke" OR "ischemic stroke" OR "hemorrhagic stroke") AND ("high-intensity interval training" OR HIIT OR "interval training" OR "high-intensity training") AND ("randomized controlled trial" OR random* OR trial OR controlled) AND ("Scandinavian Stroke Scale" OR SSS OR NIHSS OR "stroke severity")) AND (LIMIT-TO(LANGUAGE, "English")) |
| Scopus | E7 | TITLE-ABS-KEY((stroke OR "cerebrovascular accident" OR CVA OR poststroke OR "post-stroke" OR "ischemic stroke" OR "hemorrhagic stroke") AND ("high-intensity interval training" OR HIIT OR "interval training" OR "high-intensity training") AND ("randomized controlled trial" OR random* OR trial OR controlled) AND (mobility OR gait OR walking OR locomotion)) AND (LIMIT-TO(LANGUAGE, "English")) |
| Cochrane Library | E1 | ((stroke OR "cerebrovascular accident" OR CVA OR poststroke OR "post-stroke" OR "ischemic stroke" OR "hemorrhagic stroke") AND ("high-intensity interval training" OR HIIT OR "interval training" OR "high-intensity training") AND ("randomized controlled trial" OR random* OR trial OR controlled) AND ("Berg Balance Scale" OR BBS OR balance)) in Title Abstract Keyword |
| Cochrane Library | E2 | ((stroke OR "cerebrovascular accident" OR CVA OR poststroke OR "post-stroke" OR "ischemic stroke" OR "hemorrhagic stroke") AND ("high-intensity interval training" OR HIIT OR "interval training" OR "high-intensity training") AND ("randomized controlled trial" OR random* OR trial OR controlled) AND ("6-minute walk" OR 6MWT)) in Title Abstract Keyword |
| Cochrane Library | E3 | ((stroke OR "cerebrovascular accident" OR CVA OR poststroke OR "post-stroke" OR "ischemic stroke" OR "hemorrhagic stroke") AND ("high-intensity interval training" OR HIIT OR "interval training" OR "high-intensity training") AND ("randomized controlled trial" OR random* OR trial OR controlled) AND ("10-meter walk" OR 10MWT OR "gait speed")) in Title Abstract Keyword |
| Cochrane Library | E4 | ((stroke OR "cerebrovascular accident" OR CVA OR poststroke OR "post-stroke" OR "ischemic stroke" OR "hemorrhagic stroke") AND ("high-intensity interval training" OR HIIT OR "interval training" OR "high-intensity training") AND ("randomized controlled trial" OR random* OR trial OR controlled) AND ("blood pressure" OR hypertension OR SBP OR DBP)) in Title Abstract Keyword |
| Cochrane Library | E5 | ((stroke OR "cerebrovascular accident" OR CVA OR poststroke OR "post-stroke" OR "ischemic stroke" OR "hemorrhagic stroke") AND ("high-intensity interval training" OR HIIT OR "interval training" OR "high-intensity training") AND ("randomized controlled trial" OR random* OR trial OR controlled) AND ("cardiorespiratory fitness" OR VO2peak OR VO2max OR "peak oxygen uptake")) in Title Abstract Keyword |
| Cochrane Library | E6 | ((stroke OR "cerebrovascular accident" OR CVA OR poststroke OR "post-stroke" OR "ischemic stroke" OR "hemorrhagic stroke") AND ("high-intensity interval training" OR HIIT OR "interval training" OR "high-intensity training") AND ("randomized controlled trial" OR random* OR trial OR controlled) AND ("Scandinavian Stroke Scale" OR SSS OR NIHSS OR "stroke severity")) in Title Abstract Keyword |
| Cochrane Library | E7 | ((stroke OR "cerebrovascular accident" OR CVA OR poststroke OR "post-stroke" OR "ischemic stroke" OR "hemorrhagic stroke") AND ("high-intensity interval training" OR HIIT OR "interval training" OR "high-intensity training") AND ("randomized controlled trial" OR random* OR trial OR controlled) AND (mobility OR gait OR walking OR locomotion)) in Title Abstract Keyword |

Note: The above strategies show the concept combinations used for each query (E1–E7). In each database, field tags and controlled vocabulary were applied as available, and the syntax was adapted accordingly. The search covered database inception to December 31, 2025 and was limited to English-language records.
